# Adaptive smartphone-based sensor fusion for estimating competitive rowing kinematic metrics

**DOI:** 10.1371/journal.pone.0225690

**Published:** 2019-12-05

**Authors:** Bryn Cloud, Britt Tarien, Ada Liu, Thomas Shedd, Xinfan Lin, Mont Hubbard, R. Paul Crawford, Jason K. Moore

**Affiliations:** 1 Mechanical and Aerospace Engineering, University of California Davis, Davis, California, United States of America; 2 Hegemony Technologies LLC, Davis, California, United States of America; King Abdullah University of Science and Technology, SAUDI ARABIA

## Abstract

Competitive rowing highly values boat position and velocity data for real-time feedback during training, racing and post-training analysis. The ubiquity of smartphones with embedded position (GPS) and motion (accelerometer) sensors motivates their possible use in these tasks. In this paper, we investigate the use of two real-time digital filters to achieve highly accurate yet reasonably priced measurements of boat speed and distance traveled. Both filters combine acceleration and location data to estimate boat distance and speed; the first using a complementary frequency response-based filter technique, the second with a Kalman filter formalism that includes adaptive, real-time estimates of effective accelerometer bias. The estimates of distance and speed from both filters were validated and compared with accurate reference data from a differential GPS system with better than 1 cm precision and a 5 Hz update rate, in experiments using two subjects (an experienced club-level rower and an elite rower) in two different boats on a 300 m course. Compared with single channel (smartphone GPS only) measures of distance and speed, the complementary filter improved the accuracy and precision of boat speed, boat distance traveled, and distance per stroke by 44%, 42%, and 73%, respectively, while the Kalman filter improved the accuracy and precision of boat speed, boat distance traveled, and distance per stroke by 48%, 22%, and 82%, respectively. Both filters demonstrate promise as general purpose methods to substantially improve estimates of important rowing performance metrics.

## Introduction

Non-intrusive collection of data from athletes during practice and competition provides opportunities for evidenced-based performance evaluation and coaching. Traditional kinematic measurement techniques in sports have frequently required elaborate equipment to capture the motion of human body segments and associated sports equipment; see examples in [1]. With the growing functionality and ubiquity of smartphones, athletes and coaches have access to an increasingly capable and sophisticated measurement system that includes the phone’s inertial measurement unit (three dimensional angular rate gyroscope, accelerometer, and magnetometer) and determinants of location (GPS, GLONASS, etc.). Modern smartphone technology provides position measurements that can be sampled up to about 1 Hz with stationary absolute accuracy between 0.5 m to 16 m and stationary root mean square error (RMSE) between 14 m to 71 m, making them more precise than accurate [2]. The phones also output acceleration and angular velocity data at rates up to about 200 Hz [3].

Competitive rowing aims at maximizing the average boat speed over a specified race distance. For competitions over a typical race distance, the time domain race-to-race variability for elite rowers is approximately 1% and this has been proposed as “an irreducible error for any measure of rowing performance” [4]. However, the discrete unit of action and control in rowing is the stroke and this accordingly represents the domain in which many training and racing parameters are communicated and analyzed. For example, rowing speed is represented in the stroke domain as the product of stroke rate and distance per stroke.

In Olympic rowing races, the historical speed difference between finish positions (first and second; second and third; etc.) has averaged at 0.42% [5]. Contextualized in the approximately 200 strokes that it takes to complete a 2000 m race, rowers who generate an additional 5 cm per stroke will ordinarily gain a one place improvement in race finish. Thus, it follows that the accuracy and precision of distance per stroke measurements must be better than 5 cm in order to generate meaningful insight and feedback. Satellite-based positioning systems (GPS, etc.) do not ordinarily afford this level of accuracy and precision thus limiting their effectiveness in the analysis of any individual stroke. We posit that more accurate and precise measures of boat movements for individual strokes will enable a more direct examination of the causal relationships between rower-oar-boat system mechanics and race performance. Therefore this study seeks to improve the accuracy and precision of rowing performance metric measurements.

The paper begins with a brief review of the immediately related literature and is followed by an explanation of the problem and statistics used to quantify accuracy and precision of the desired kinematic performance metrics. Two methods are then presented for fusing the smartphone position and motion data to generate more accurate estimates of these metrics. Finally, the estimates are presented against ground truth data collected from a differential GPS (DGPS) system for validation. We close with discussion of the implications and use cases.

## Related work

Real-time water-relative boat speed in rowing has traditionally been measured by either a pitot tube or a small impeller attached to the hull. Modern speedometers make use of GPS receivers to calculate Earth-relative speed and distance in the distance, time, and stroke domains. For example, the popular SpeedCoach GPS (Nielsen Kellerman, Boothwyn, PA, USA) outputs metrics such as boat speed, stroke rate, distance, and elapsed time based on GPS and/or impeller measurements. The accuracy and utility of these systems are limited by the position measurement accuracy and/or the uncertain and frequently fluctuating current velocity. GPS alone has been used to measure position during long distance (15,000 m) rowing events [6] and low cost GPS systems have also been shown to be capable of providing real-time speed estimates during rowing [7].

Other references exist with high accuracy (0.1 ms^-1^ to 0.3 ms^-1^) GPS measurements for rowing [8] and the use of high accuracy differential GPS [9], but these systems are often impractical for ordinary rowing applications because they require establishing and operating an additional stationary base station. There has been success in creating differential GPS systems from a network of smartphones that improve location estimates to 1 cm accuracy at 1 Hz [10] and a differential GPS-tailored Kalman filter has been used for the specific task of rowing position prediction [11].

Researchers have improved the accuracy of position and speed estimates in rowing by incorporating acceleration measures. Accelerometer-derived speed shows strong correlation to impeller-derived speed measurements in still water [12]. GPS and accelerometer sensor fusion have been used to estimate position and velocity during GPS network downtime [13, 14]. Reference [15] compares GPS accelerometer-derived velocity to high speed video footage, and [16] measures differential GPS and acceleration showing the utility of advanced sensors.

A network of IMUs on the rower can capture rowing with results similar to motion capture cameras [17] and real-time accelerometer-based feedback has been found to improve rowing consistency when used on indoor ergometers [18]. Tessendorf et. al [19] use an elaborate IMU sensor array (Xsens, Enschede, Netherlands) to demonstrate the utility of metrics for characterizing on-water rowing performance but this system requires extensive setup time and expertise and is cost prohibitive for the typical rower. Various filters have been used to improve smartphone position estimates for walking in [20], but the large sensor error causes difficulties when applied to this more general problem.

Among the various methods that have been proposed to improve measurement results during rowing, the most similar to the present paper is that of Hermsen [21]. Hermsen’s primary goal was to estimate the position, speed, and stroke rate of the boat based on a consumer-grade accelerometer and GPS sensor for real-time wireless transmission and display to viewers of the rowing event. The proposed linear Kalman filter-based approach fused data from the two sensors and estimated rowing speed. The found finish times are 14% more accurate than those estimated with GPS data alone. Although real-time estimates were desired, his solution to handling sensor orientation bias required an offline after-the-fact computation leaving real-time implementation infeasible.

None of these prior methods offer an accurate and precise estimate of boat distance traveled and boat speed that is inexpensive, simple, works with a single consumer grade GPS sensor, and can operate in real-time. In this paper, we present two methods that can do so. These methods provide a strong foundation for further improvements to the desired estimates.

## Problem formulation

We desire highly accurate estimates of the distance the boat travels along its path during each individual stroke using readily available and easy to use consumer products, such as, a smartphone. High accuracy allows for inter-rower, -race, and -day repeatable comparisons in both distance traveled and boat speed. In competitive rowing, boats move on the order of 10 m per stroke. We have found smartphones to have raw accuracy on the order of 1 m and a precision of 0.8 m by comparison with our differential GPS measurements; see Table 1. Our ultimate goal is to improve this distance accuracy by roughly two orders of magnitude, allowing distance per stroke estimates that approach 1 cm accuracy. Additionally, we want the capability of calculating these estimates in real time and to not rely on knowledge of the specific boat and rower to facilitate easy to use and simple real-time training feedback to coaches and rowers. Our proposed methods to accomplish these goals consist of four major components:

**Data collection** A smartphone is rigidly attached to a boat and used to collect GPS data at an average sampling rate of 0.3 Hz and accelerometer data at approximately 100 Hz. (A differential GPS unit is also attached to the boat to measure boat position at approximately 5 Hz for validation purposes, but this is not part of the evaluated method).
**Sensor fusion** Fusion of the raw GPS and accelerometer measurements to estimate distance traveled at the accelerometer sampling rate (100 Hz).
**Rowing metric computation** Stroke transition detection is used to calculate the distance traveled per stroke, stroke rate, and boat speed.
**Error estimates** Estimates from the sensor fusion are compared to “true” values obtained from the differential GPS measurements.

**Table 1:** Sensor measurement accuracy and precision. The rows corresponding to the smartphone GPS provide the accuracy (central error, CE [2]) and precision (standard deviation, SD) of the GPS-derived position relative to simultaneously collected DGPS position of the moving pair of sensors (see the following section for our definitions of these statistics). The smartphone accelerometer rows provide a measure of precision of the sensor’s body fixed acceleration when the smartphone is motionless. Similarly, the differential GPS rows provide a measure of precision of the motionless rower position relative to the motionless base station. The duration of the data logs used to derive these metrics and the frequency at which they were sampled are listed for each sensor.

Sensor	Measurement	Value
Smartphone GPS (Moving, 32 sec, 0.3 Hz)	CE of NS position	1.01 m
	CE of EW position	0.89 m
	SD in the NS position	0.81 m
	SD in the EW position	0.70 m
Smartphone Accelerometer (Motionless, 96 sec, 100 Hz)	SD along the X axis	2.67 mg
	SD along the Y axis	2.45 mg
	SD along the Z axis	1.59 mg
Differential GPS (Motionless, 57 sec, 10 Hz)	SD in the N-S position	3.2 mm
	SD in the E-W position	1.7 mm

**Fig 1:**
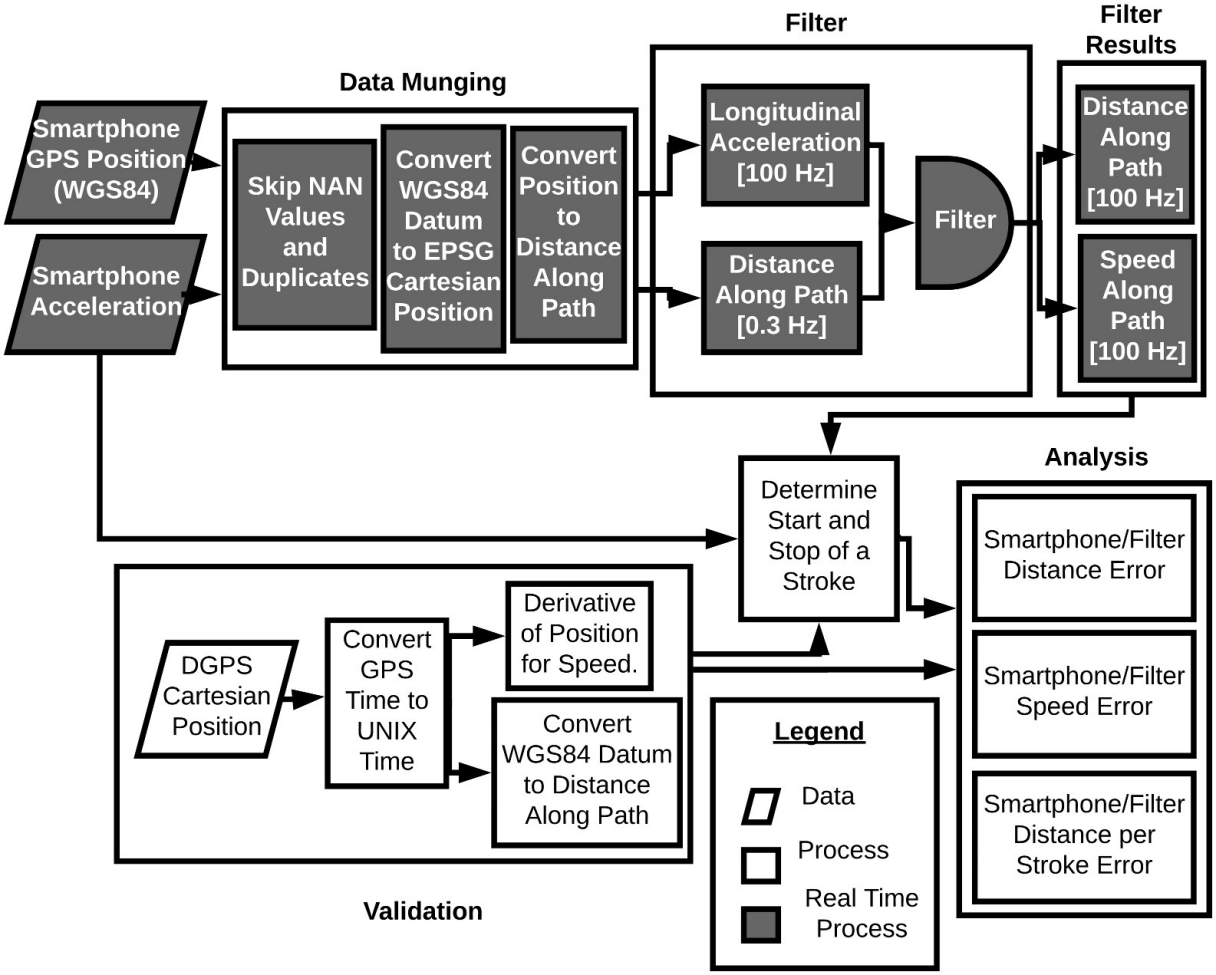
Data processing pipeline flow chart. Grey rectangles indicate the real-time algorithm process. White rectangles indicate the validation process. Parallelograms represent raw data from the sensors.

Fig 1 provides a schematic of the aforementioned general flow of data and processing algorithms. The primary algorithms, i.e. transforming raw smartphone data to distance and speed estimates, are designed for real-time computing, but the actual results for the purposes of the paper were computed offline and are available in the companion software (see https://gitlab.com/mechmotum/row_filter). In this section we elaborate on the four components listed above, beginning with the characterization of the measurement data. We then propose the desired accuracy of the metrics, and finally provide the details of the two sensor fusion methods.

### Accuracy and precision

It is worth carefully defining the accuracy and precision of repeated measurements of a motionless sensor and those of a moving sensor [2].

*Accuracy* specifies how close a given measurement is to the true value. In the case of planar Cartesian horizontal position measurements (*x*_1_…*x*_*n*_, *y*_1_…*y*_*n*_) derived from latitude and longitude of a motionless sensor, we use the Central Error, CE, defined in [2] as a measure of accuracy. This is the Euclidean distance between the average of a set of measurements, $\left(\bar{x},\bar{y}\right)$, and the sensor’s true position, (*x*_*s*_, *y*_*s*_).

$${\text{CE}}_{xy}^{2}={\left[\frac{1}{n}\sum_{i=1}^{n}\left(x_{i}-x_{s}\right)\right]}^{2}+{\left[\frac{1}{n}\sum_{i=1}^{n}\left(y_{i}-y_{s}\right)\right]}^{2}={\left(\bar{x}-x_{s}\right)}^{2}+{\left(\bar{y}-y_{s}\right)}^{2}$$ (1)

*Precision* characterizes how repeatable measurements are. For measurements from a motionless sensor the standard deviation, *σ*, about the mean position in the planar Cartesian coordinates is a measure of precision.

$$\sigma_{x}^{2}=\frac{1}{n}\sum_{i=1}^{n}{\left(x_{i}-\bar{x}\right)}^{2},\;\sigma_{y}^{2}=\frac{1}{n}\sum_{i=1}^{n}{\left(y_{i}-\bar{y}\right)}^{2}$$ (2)

The Federal Geographic Data Committee recommends using the Root Mean Square Error (RMSE) to characterize error in geographic position measurements [2]. It is important to note that RMSE is a function of both accuracy and precision. For example, increases in either the Central Error or the standard deviation will increase RMSE:

$${\text{RMSE}}_{xy}^{2}=\frac{1}{n}\sum_{i=1}^{n}[{\left(x_{i}-x_{s}\right)}^{2}+{\left(y_{i}-y_{s}\right)}^{2}]={\text{CE}}_{xy}^{2}+\frac{n-1}{n}(\sigma_{x}^{2}+\sigma_{y}^{2})$$ (3)

We have elected to report RMSE values in this paper to follow this convention. We calculate the error between the smartphone measurements (or smartphone derived estimates) and the measurements from the differential GPS, which we define as ground truth.

Furthermore, we are primarily concerned with estimates of the distance, *d*(*t*), and speed, *v*(*t*), along the boat’s nearly straight path during rowing. So we additionally define the accuracy and precision of these time varying estimates. We calculate the distance for the smartphone, *d*_SP_, and DGPS, *d*_DGPS_, at any given discrete time measurement, *t*_*i*_, with the following equation, using smartphone or DGPS data respectively:

$$d\left(t_{i}\right)=d\left(t_{i-1}\right)+\sqrt{{\left[x\left(t_{i}\right)-x\left(t_{i-1}\right)\right]}^{2}+{\left[y\left(t_{i}\right)-y\left(t_{i-1}\right)\right]}^{2}}.$$ (4)

The boat speed is then estimated from the DGPS data using backward differences.

$$v_{\text{DGPS}}\left(t_{i}\right)=\frac{d_{\text{DGPS}}\left(t_{i}\right)-d_{\text{DGPS}}\left(t_{i-1}\right)}{t_{i}-t_{i-1}}$$ (5)

Additionally, the boat speed is also reported directly from the smartphones internal estimates.

Given the boat distance and speed along the path we calculate the RMSE of any estimate of the two prior quantities by comparing them with the counterparts derived from the differential GPS data to quantify accuracy and precision (Eqs (6) and (7)). In this case *n* is taken as the number of samples associated with the signal of higher sampling rate, and linear interpolation is used to find intermediate samples of the signal with lower sampling rate.

$${\text{RMSE}}_{d}^{2}=\frac{1}{n}\sum_{i=1}^{n}d_{e}{\left(t_{i}\right)}^{2}=\frac{1}{n}\sum_{i=1}^{n}{\left[d\left(t_{i}\right)-d_{\text{DGPS}}\left(t_{i}\right)\right]}^{2}$$ (6)

$${\text{RMSE}}_{v}^{2}=\frac{1}{n}\sum_{i=1}^{n}v_{e}{\left(t_{i}\right)}^{2}=\frac{1}{n}\sum_{i=1}^{n}{\left[v\left(t_{i}\right)-v_{\text{DGPS}}\left(t_{i}\right)\right]}^{2}$$ (7)

With given errors *d*_*e*_ and *v*_*e*_ at every time sample, the mean of the errors (Eqs (8) and (9)), and the standard deviation of the errors, (Eqs (10) and (11)) can be computed with

$${\bar{d}}_{e}=\frac{1}{n}\sum_{i=1}^{n}d_{e}\left(t_{i}\right)=\frac{1}{n}\sum_{i=1}^{n}[d\left(t_{i}\right)-d_{\text{DGPS}}\left(t_{i}\right)]$$ (8)

$${\bar{v}}_{e}=\frac{1}{n}\sum_{i=1}^{n}v_{e}\left(t_{i}\right)=\frac{1}{n}\sum_{i=1}^{n}[v\left(t_{i}\right)-v_{\text{DGPS}}\left(t_{i}\right)]$$ (9)

$$\sigma_{d_{e}}^{2}=\frac{1}{n}\sum_{i=1}^{n}{\left[d_{e}\left(t_{i}\right)-{\bar{d}}_{e}\right]}^{2}$$ (10)

$$\sigma_{v_{e}}^{2}=\frac{1}{n}\sum_{i=1}^{n}{\left[v_{e}\left(t_{i}\right)-{\bar{v}}_{e}\right]}^{2}$$ (11)

The central errors are then simply

$${\text{CE}}_{d_{e}}=\sqrt{{\bar{d}}_{e}^{2}}=|{\bar{d}}_{e}|,\;{\text{CE}}_{v_{e}}=\sqrt{{\bar{v}}_{e}^{2}}=\left|{\bar{v}}_{e}\right|.$$ (12)

The RMSE is related to the error mean and standard deviation by

$${\text{RMSE}}_{d}^{2}={\text{CE}}_{d_{e}}^{2}+\sigma_{d_{e}}^{2},\;{\text{RMSE}}_{v}^{2}={\text{CE}}_{v_{e}}^{2}+\sigma_{v_{e}}^{2}.$$ (13)

Lastly, we calculate the RMSE of the actual distance per stroke relative to the estimated distance per stroke for all strokes, or subsets of strokes.

$${\text{RMSE}}_{d_{s}}=\sqrt{\frac{\sum_{i=1}^{m}{\left[d_{si}-d_{\text{DGPS}si}\right]}^{2}}{m}}$$ (14)

where *d*_*si*_ is the ith distance per stroke from an estimate and *m* is the number of strokes.

### Data collection

#### Smartphone GPS

The smartphone provides global position estimates accessed via the iPhone software development kit. Latitude and longitude are received at a variable sampling rate between 0.1 and 1 Hz, usually at an average of about 0.3 Hz when the sensor is in motion. Once the data is transformed into an Earth-local Cartesian coordinate system with respect to the WGS84 coordinate system [22], the precision of motionless measurements can be determined; see Table 1. For repeated measurements over a short duration (<15 min) we assume that any inherent systematic bias of the GPS relative to true position is constant and does not degrade our distance calculations. None of the metrics of interest we describe later requires knowledge of the absolute position of the boat on the earth; instead we require only relative sample-to-sample position differences. Even though systematic bias can be quite large, e.g. 16 m, the precision of repeated measurements over a short duration can be at least an order of magnitude lower [2], which is advantageous in our case.

Using a Piksi differential GPS system (SwiftNav, San Francisco, USA) as a measure of ground truth relative position (with better than 1 cm precision) we characterized the motionless and moving mean-subtracted distribution of smartphone position measurement errors; see Table 1. The cumulative distance traveled along the boat’s path is calculated from the relative distance between each (*x*, *y*) coordinate; see Eq (4). We rely on numerical differentiation (backward differences, see Eq (5)) using the sensor-recorded time stamps to compute speed from the DGPS position measurements.

#### Smartphone acceleration

The smartphone accelerometer provides three dimensional body-fixed acceleration measurements with an average precision (SD) of about 0.02 ms^-2^, updated at approximately 100 Hz. When affixed to the boat, we are interested in the component of acceleration tangent to the boat’s travel path on the water surface, which is approximately the smartphone’s *y* component in our case.

**Fig 2:**
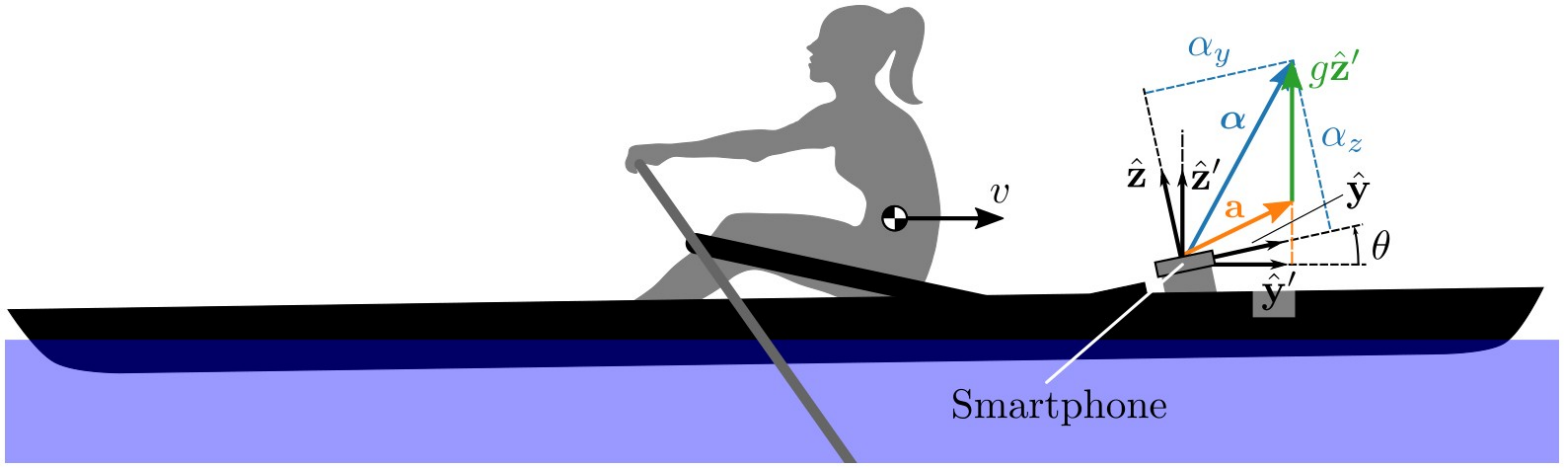
Diagram of a rowing boat under speed with an imperfectly aligned bow-mounted smartphone. The smartphone coordinate system $\hat{y},\hat{z}$ is oriented relative to the horizontal coordinate system ${\hat{y}}^{\prime },{\hat{z}}^{\prime }$ by a varying pitch angle *θ*. The accelerometer-reported acceleration **α** differs from the actual ${\hat{y}}^{\prime }$ component because it includes a gravitational component. The actual acceleration **a** of the phone is then $a=\alpha -g{\hat{z}}^{\prime }$. We desire the magnitude of the acceleration **a** projected onto the horizontal plane but we do not know *θ* at any given time. As described in the “smartphone acceleration” section, the sensed $\hat{y}$ acceleration differs from the true ${\hat{y}}^{\prime }$ acceleration by a moderate intrinsic bias and a larger term *gθ* which accounts for the projection of the gravity vector on the pitched $\hat{y}$ axis.

The small yaw (typically <1°) angular motion during typical rowing [5] allows us to ignore the lateral acceleration component. We also ignore effects of any boat rolling motion, because it is typically negligible as well [5]. Pitch angular motion is similarly small (<1°) [5] but because of the relatively large gravitational acceleration, even small changes in pitch mounting orientation, or static boat pitch mean that the longitudinal smartphone acceleration measurement will be biased; see Fig 2.

In general, we use only the smartphone-fixed longitudinal component of acceleration, *α*_*y*_ to estimate distance, but must take into account the pitch effects and accumulation of error from twice integrating the biased accelerometer measurement. Although this could be corrected by a calibration procedure [21], it is generally not practical in the expected smartphone consumer use case. Fig 2 illustrates how the smartphone body-fixed sensed acceleration relates to the actual acceleration parallel to the water’s surface. The acceleration vector $a=\alpha -g{\hat{z}}^{\prime }$ can be written as two scalar equations by projecting onto the ${\hat{y}}^{\prime },{\hat{z}}^{\prime }$ axes.

$$a_{y}=\alpha_{y}\text{cos}\theta -\alpha_{z}\text{sin}\theta$$ (15)

$$a_{z}=\alpha_{y}\text{sin}\theta -\alpha_{z}\text{cos}\theta -g$$ (16)

These two equations can be combined to show that the longitudinal acceleration is:

$$a_{y}=\frac{\alpha_{y}}{\text{cos}\theta }-\left(a_{z}+g\right)\text{tan}\theta$$ (17)

If the smartphone pitch, *θ*, and the vertical acceleration, *a*_*z*_, of the boat are small, then the longitudinal acceleration *a* is given by the following linear approximation:

$$a=a_{y}\approx \alpha_{y}-g\theta .$$ (18)

For example, if *θ* were 6 degrees due to off-level mounting and average boat pitch, the gravity term could cause up to a meter per second squared error in the estimate.

### Desired kinematic metrics

#### Stroke rate

**Fig 3:**
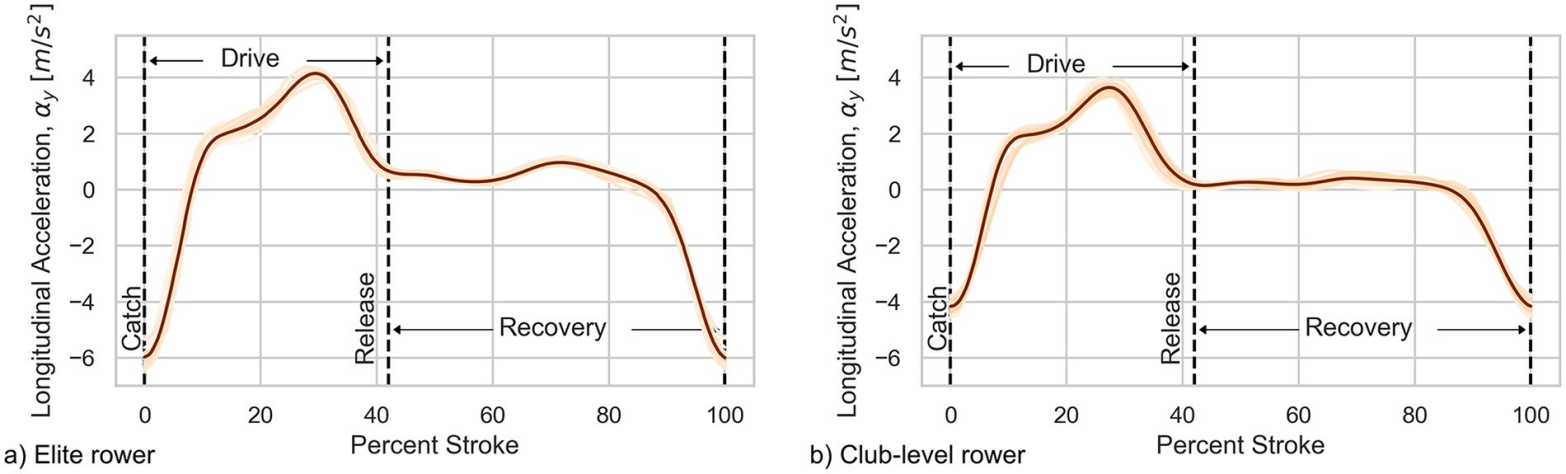
Boat-fixed longitudinal acceleration as a function of percent stroke from two trials (elite and club-level 24SE.). Each stroke is plotted as an orange line and the mean of all strokes as a dark line. The repeatability of the measured longitudinal acceleration, especially for the elite rower, validates the consistency of rowing technique, the robustness of the stroke endpoint identification, and the quality of the accelerometer data itself.

Rowing involves periodic propulsive strokes by the rower(s) delivered through the oars to generate boat movement. These create a periodic kinematic pattern of boat accelerations and pitching that reliably maps to the characteristic phases of the stroke. Similar to others [14], we defined the endpoints of the stroke (the end of one and start of the next) as the timepoint that corresponds to the minimum peak values of longitudinal boat acceleration. This instant in time reliably corresponds to the transition from the recovery phase to the beginning of the propulsive phase of the stroke, commonly referenced in rowing as the “catch” [5]. These time instants can be detected in real-time using the method from [23], for example. Fig 3 illustrates the reliability of individual stroke endpoints detected using this method as well as the consistency of the rowing technique and the data quality during the experiments. On the rare occasion when visual inspection of the data demonstrated a clear stroke detection misidentification, the data from that stroke was excluded from any relevant analyses. These stroke timepoints are then used to calculate the stroke-domain metrics of interest: distance per stroke and stroke rate.

#### Boat speed

**Fig 4:**
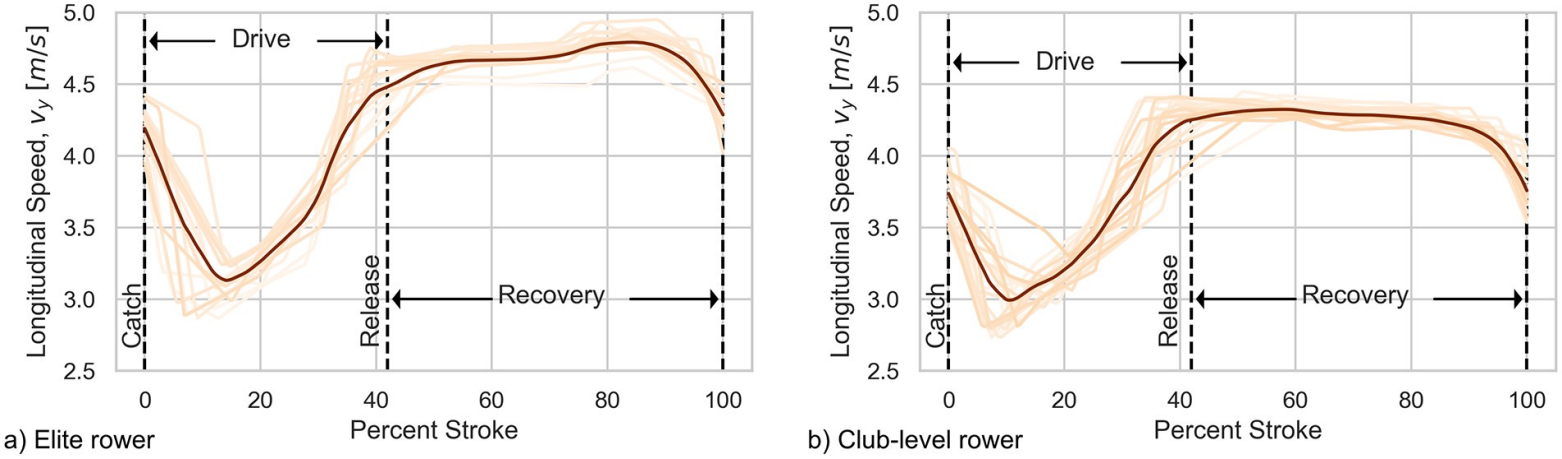
DGPS computed boat speed as a function of percent stroke for two trials (elite and club-level 24SE). Each stroke is plotted as an orange line and the mean of all strokes as a dark line. Because the DGPS data is sampled at only 5 Hz and the relative precision is lower than the accelerometer, the measured speed profile is less smooth and repeatable than the acceleration profiles in Fig 3.

Average boat speed along the shortest path to the finish is the primary metric rowers must maximize to win a race. We can compute reference boat speed by using the differential GPS measurements and Eq (5), and for the smartphone we rely on its internal speed estimate directly as it seems to be estimated via an algorithm that is more accurate than simple numerical differentiation of the distance. Fig 4 shows the DGPS computed speed measures for two trials at the same stroke rate.

Determining instantaneous earth-relative boat speed relies on accurate distance estimates. The smartphone provides a moderately accurate but reasonably precise position update at a sample rate on the same order of magnitude as the stroke rate, i.e. 0.3 Hz, which is only useful for average speed estimates over a number of strokes. Given a 0.8m precision in the distance measurements (Table 1), the accuracy of the speed estimates from the phone are on the order of 0.3 ms^-1^. If the desired location precision of 5 cm was achieved at sampling rates approaching 100 Hz, the speed accuracy and frequency updates could potentially increase to 0.02 ms^-1^ and a thus deliver data on intra-stroke speed variations.

#### Distance per stroke

Boat speed is the product of two separate but correlated variables in the stroke domain: stroke rate and distance per stroke. We calculate distance per stroke for each stroke by subtracting the interpolated distance, Eq (4), at each pair of subsequent stroke start/stop times. The same synchronized start/end time values are used for the smartphone-derived and reference differential GPS data allowing a direct comparison of the various estimations of boat distance. This comparison will allow an estimate of the accuracy and precision of each estimate method presented below.

As another indication of the effectiveness of the stroke endpoint identification procedure and the subsequent calculations of stroke time, distance per stroke and average speed, Table 2 reports the derived data and statistics on the strokes of the two trials portrayed in Figs 3 and 4.

**Table 2:** Summary DGPS data for strokes from elite and club-level 24SE trials depicted in Figs 3 and 4.

	elite			club-level		
Stroke	Duration [s]	Distance [m]	Speed [m s^−1^]	Duration [s]	Distance [m]	Speed [m s^−1^]
1	2.52	10.10	4.03	2.50	9.80	3.90
2	2.48	10.10	4.05	2.43	9.43	3.80
3	2.37	9.89	4.15	2.44	9.71	3.98
4	2.36	9.98	4.18	2.37	9.49	4.02
5	2.35	10.03	4.26	2.52	10.02	3.93
6	2.31	9.79	4.27	2.62	10.28	3.95
7	2.29	9.89	4.32	2.37	9.42	3.95
8	2.33	10.06	4.30	2.41	9.59	3.99
9	2.25	9.59	4.22	2.49	9.82	3.91
10	2.28	9.84	4.39	2.49	9.84	3.86
11	2.28	9.89	4.36	2.47	9.63	3.90
12	2.43	10.52	4.36	2.37	9.32	3.95
13	2.41	10.36	4.26	2.57	10.07	3.90
14	2.40	10.27	4.34	2.44	9.66	3.93
15	2.39	10.21	4.32	2.61	10.34	3.97
16	2.42	10.20	4.24	2.43	9.47	3.93
17	2.37	9.98	4.20	2.47	9.76	3.92
18	2.38	10.09	4.24	2.46	9.68	3.96
19	2.34	9.95	4.20	2.46	9.56	3.86
20	2.33	9.66	4.16	2.56	9.98	3.92
21				2.42	9.34	3.91
22				2.43	9.54	3.97
23				2.46	9.62	3.93
24				2.44	9.30	3.78
AVG	2.36	10.02	4.24	2.46	9.69	3.92
STD	0.07	0.23	0.10	0.07	0.29	0.05

### Sensor fusion method 1: Complementary filter

**Fig 5:**
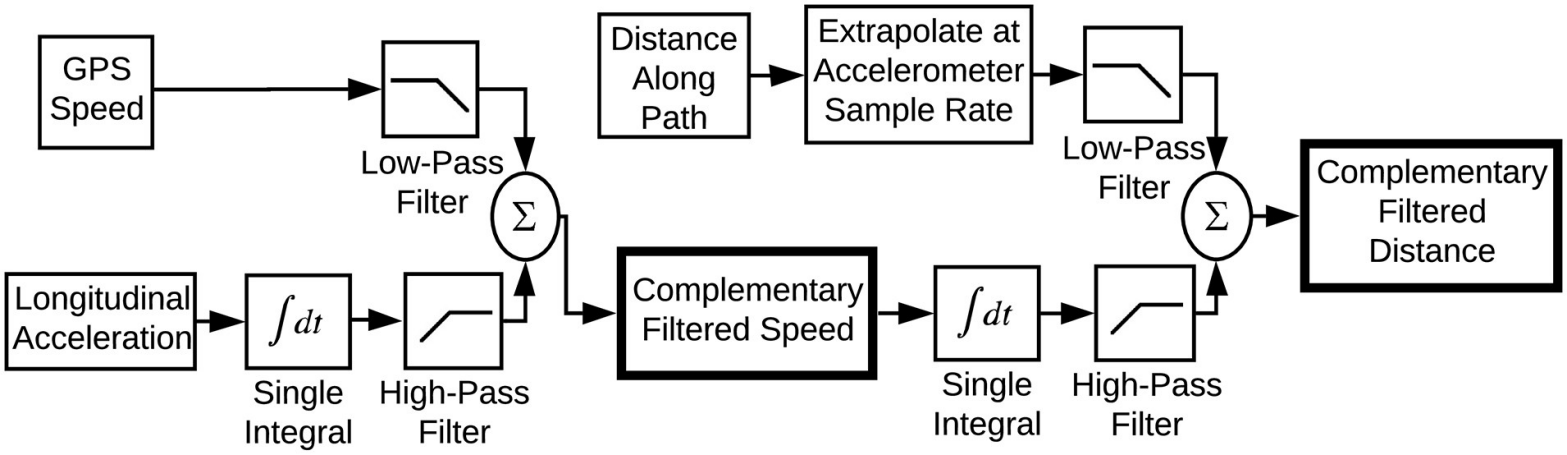
Block diagram depicting the complementary filter algorithm. The smartphone speed and acceleration are fused to create an improved speed estimate and then that estimate is fused with the smartphone GPS-derived distance to create an improved distance estimate.

The first method of combining smartphone accelerometer and GPS data stems from the classical idea of characterizing input-output behavior based on frequency response. We utilize two complementary filters in series, Fig 5, with each filter made up of two real-time discrete 2^nd^ order Butterworth filters, namely one low-pass presented in [24] and one high-pass of similar design. Integrating the biased and noisy acceleration measurement introduces drift in the resulting speed and distance estimates, as expected. The high-pass filter is used to extract the high frequency portion of these estimates and to exclude the low frequency drift component. The low-pass filter extracts the low frequency portion of the smartphone speed and GPS-derived distance estimates. Each pair of two filtered signals is then summed at each accelerometer sample time to update the estimates. The results are more accurate speed and distance estimates.

#### Extrapolating GPS data

Since the GPS measurements occur less frequently than the accelerometer measurements, the smartphone speed and GPS-derived distance are linearly extrapolated, Eq (19), between GPS updates at each accelerometer update. This process uses the prior two GPS samples to provide a smoothed complementary filter input. This simple additional “filtering” procedure improves the distance estimate by 37% and the speed estimate by 20%. In the equation below the *i* index represents the accelerometer update time and the *k* index represents the last GPS update prior to *t*_*i*_. This amounts to using the average speed derived from the GPS to make the extrapolation.

$$d(t_{i})=d\left(t_{i-1}\right)+\frac{d\left(t_{k}\right)-d\left(t_{k-1}\right)}{t_{k}-t_{k-1}}\left(t_{i}-t_{i-1}\right)$$ (19)

#### Bias and the Butterworth filter

A Butterworth filter creates a maximally flat passband and is relatively easy to implement digitally. At the cutoff frequency it rolls off gradually but is sufficient for many biomechanical filtering needs [1]. The transfer functions for the low- and high-pass 2^nd^ order Butterworth filters are shown in Eqs (20) and (21) together with the equations for the magnitudes of frequency response.

$$H_{\text{low}}\left(s\right)=\frac{\omega_{c}}{s^{2}+\sqrt{2}\omega_{c}s+\omega_{c}},\;\left|H_{\text{low}}\left(j\omega \right)\right|=\frac{1}{\sqrt{1+{\left(\frac{\omega }{\omega_{c}}\right)}^{4}}}$$ (20)

$$H_{\text{high}}\left(s\right)=\frac{s^{2}}{s^{2}+\sqrt{2}\omega_{c}s+\omega_{c}},\;\left|H_{\text{high}}\left(j\omega \right)\right|=\frac{1}{\sqrt{1+{\left(\frac{\omega_{c}}{\omega }\right)}^{4}}}$$ (21)

In the first filter in this series, we low-pass filter the smartphone GPS speed estimate and high-pass filter the longitudinal accelerometer measurement. The accelerometer output is the sum of the acceleration along the travel path minus a term that varies with boat pitch around a constant bias, i.e. *α*_*y*_ − *gθ*. In the frequency domain, these two input signals can be written as

$$X_{\text{low}}\left(j\omega \right)=V(j\omega )$$ (22)

$$X_{\text{high}}\left(j\omega \right)=A\left(j\omega \right)+a_{0}$$ (23)

where *a*_0_ represents the bias. Once filtered, the magnitude of each signal becomes

$$|Y_{low}\left(jw\right)|=\frac{V(jw)}{\sqrt{1+{\left(\frac{w}{w_{c}}\right)}^{4}}}$$ (24)

$$|Y_{high}\left(jw\right)|=\frac{\left|A(jw)\right|}{\sqrt{1+{\left(\frac{w_{c}}{w}\right)}^{4}}}+\frac{a_{0}}{\sqrt{1+{\left(\frac{w_{c}}{w}\right)}^{4}}}.$$ (25)

**Fig 6:**
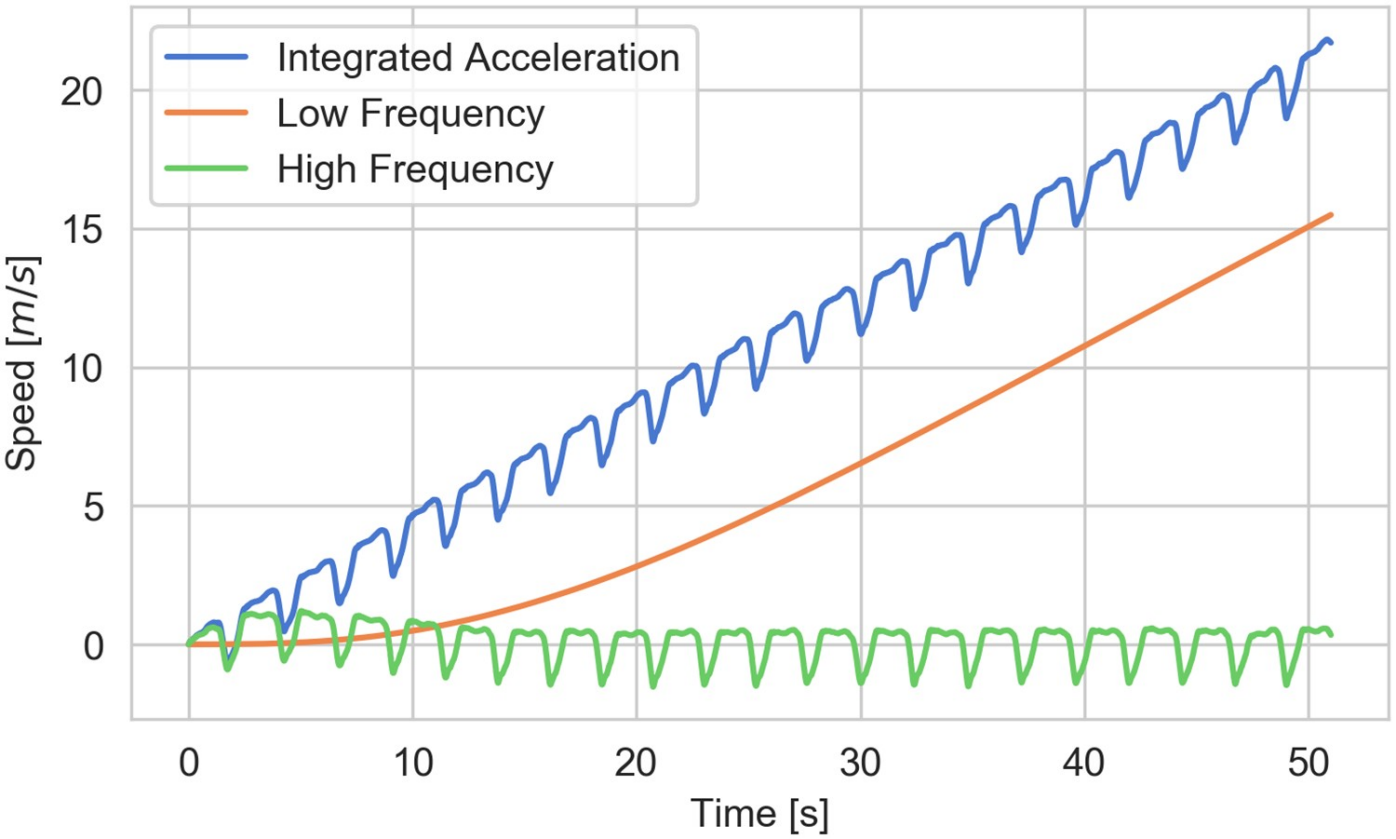
Example of low and high frequency components of speed derived from the integrated accelerometer measurement. The blue curve shows the effect of the accelerometer bias on the speed estimate. The approximately constant slope of this curve indicates that the accelerometer bias is also approximately constant. The orange and green curves show the measurement separated into low and high frequency components.

In order to filter the effects of the accelerometer bias from the speed estimate, the high-pass cutoff frequency must be tuned to maximize the desired signal and to minimize the bias term in Eq (25). If the bias term has significant frequency content in the same bandwidth as the desirable signal, it is difficult to separate them. Fig 6 shows that the frequency content of the bias term, *a*_0_, is very low and that the high pass filter is effective at removing the bias. Thus, this filter is very suitable for this application where the accelerometer bias is approximately constant.

#### Cutoff frequency selection

A unique cutoff frequency is computed for each filter (low-pass and high-pass, each for distance and speed) for each trial. We calculate these parameters using an offline nonlinear least squares procedure to minimize the squared error between the filtered distance and the differential GPS distance. These optimal cutoff frequencies are averaged across all trials and the result is used in the real-time implementation, see Table 3. Since the high-pass cutoff frequencies for distance were large and did not have much effect on the performance of the filter above 10 Hz, the average was computed only over cutoff frequencies below 10 Hz.

**Table 3:** Average optimal Butterworth filter cutoff frequencies.

Distance Filter		Speed Filter	
low [Hz]	high [Hz]	low [Hz]	high [Hz]
0.189	3.789	0.0166	0.0457

Using the optimal cutoff frequency for each trial as opposed to the average over all trials would decrease the distance RMSE by an average of 20% and the speed RMSE by an average of 8%. However, calculating optimal cutoff frequencies would require post-processing and would render this filter unsuitable for real-time estimation.

### Sensor fusion method 2: Kalman filter

The Kalman filter algorithm fuses data collected from different sensors with a predictive dynamic physical model to estimate the target time-varying variables of interest, known as states. The estimation results are expected to be more accurate than those obtained from any individual sensor [25]. Although the Kalman filter formalism makes several fundamental mathematical assumptions, perhaps the most important of which are random Gaussian process and measurement noise to guarantee the ultimate optimality, these assumptions are often relaxed in practice and the technique still works [26].

In our case, the body-fixed longitudinal acceleration of the boat is measured and used as an input to a kinematic model to predict the displacement and speed of the boat along its path. The predictions are then compared with the smartphone GPS-derived distance traveled and speed measurement, and the errors are used as feedback to adjust the estimation in real time. The Kalman filter gain can be tuned to balance the sensor and model uncertainty to achieve optimal accuracy. Details regarding the application of Kalman filtering to this estimation problem will be discussed in this section.

#### Boat kinematic model

The Kalman filter relies on a discrete dynamic model describing the kinematic relationships along the path. The actual horizontal acceleration *a* is integrated twice in discrete time to obtain distance *d* and speed *v*,

$$d_{k+1}=d_{k}+v_{k}\Delta t.$$ (26)

$$v_{k+1}=v_{k}+a_{k}\Delta t$$ (27)

where the subscripts are shorthand for *d*_*k*_ = *d*(*t*_*k*_), etc.

As noted previously and illustrated in Fig 2, the smartphone’s accelerometer axis $\hat{y}$ is not, in general, perfectly aligned with the boat’s horizontal travel path. Additionally, the accelerometer has an inherent bias due to its construction and nature. Neither are stationary but can be modeled as such for improved filter performance. If we want to use the smartphone acceleration *α*_*y*_ in place of *a* in Eq (27), we must compensate for these biases adjusting the accelerometer’s measurement. To do so, we introduce an unknown constant bias state, *ϕ*_*k*_, as

$$\phi_{k+1}=\phi_{k}$$ (28)

and replace *a* with *α*_*y*,*k*_ − *ϕ*_*k*_, where *ϕ*_*k*_ is a model for the sum of the inherent accelerometer bias and the mean of *gθ*(*t*) that characterizes the bias due to boat pitch from (Eq 18). The augmented speed state equation becomes

$$v_{k+1}=v_{k}+\left(\alpha_{y,k}-\phi_{k}\right)\Delta t.$$ (29)

This bias can be thought of as the “effective” bias, in that it is the sum of the “real” sensor bias and the mean value of *gθ*. It has now become a new state to be estimated by the filter which will effectively account for drift of integration error accumulation. The time varying component of *gθ*(*t*) is the sum of two parts: a roughly periodic remnant and a small truly random measurement noise. These two parts are lumped together as “process noise” **w**_*k*_ below.

Lastly, we make use of two measurements, *d* and *v*, which are the smartphone GPS derived distance and speed along the travel path to correct our kinematic model predictions. Eqs (26) and (29) can be written in state space form to facilitate the design of the filter.

$$x_{k+1}=Ax_{k}+Bu_{k}+w_{k}$$ (30)

$$y_{k}=Cx_{k}+Du_{k}+\nu_{k}$$ (31)

where

$$x_{k}={\left[d_{k},v_{k},\phi_{k}\right]}^{T},\;u_{k}=\left[\alpha_{y,k}\right],\;y_{k}={\left[d_{k},v_{k}\right]}^{T},$$ (32)

and

$$\begin{array}{c} A=[\begin{array}{ccc} 1 & \Delta t & 0\\ 0 & 1 & -\Delta t\\ 0 & 0 & 1 \end{array}],\;B=[\begin{array}{c} 0\\ \Delta t\\ 0 \end{array}],\;C=[\begin{array}{ccc} 1 & 0 & 0\\ 0 & 1 & 0 \end{array}],\;D=[\begin{array}{c} 0\\ 0 \end{array}]. \end{array}$$ (33)

The terms **w**_*k*_ and ***ν***_*k*_ are the process and measurement noise representing model and sensor uncertainty, respectively.

#### Kalman filter formulation

Based on the state space model of boat kinematics, we design a Kalman filter to estimate the states **x**_*k*_ over time. The Kalman filter generates the estimates in two steps: the model prediction update and the measurement update. In the prediction update, an *a priori* estimate is made based on the input, the estimated state at the previous time instant, and the model,

$${\hat{x}}_{k}^{-}=A{\hat{x}}_{k-1}^{+}+Bu_{k-1},$$ (34)

where the superscript ^−^ denotes the *a priori* estimate and ^+^ denotes the final (*a posteriori*) estimate. In our case, the acceleration measurement is fed as the input to the kinematic model to calculate the instantaneous speed and distance. Meanwhile, the Kalman filter provides an estimate of the covariance **P** of the state estimate according to

$$P_{k}^{-}=A_{k-1}{P^{+}}_{k-1}A_{k-1}^{T}+Q,$$ (35)

which characterizes the estimate accuracy. In Eq (35), **Q** is the assumed covariance of the process noise **w**_*k*_.

In the measurement update step, an *a posteriori* estimate is made based on the difference between the model prediction and the output measurement error feedback,

$${\hat{x}}_{k}^{+}={\hat{x}}_{k}^{-}+L_{k}\left(y_{k}-{\hat{y}}_{k}^{-}\right)$$ (36)

where

$${\hat{y}}_{k}^{-}=C_{k}{\hat{x}}_{k}^{-}+D_{k}u_{k},$$ (37)

and where **L**_*k*_ is the Kalman gain matrix calculated as

$$L_{k}=P_{k}^{-}C_{k}^{T}{\left(C_{k}P_{k}^{-}C_{k}^{T}+R\right)}^{-1}.$$ (38)

In Eq (38), **R** is the covariance of the assumed random output measurement noise **ν**_*k*_. During this step, the estimate covariance is also updated

$$P_{k}^{+}=\left(1-L_{K}C_{k}^{T}\right)P_{k}^{-}.$$ (39)

If both the process noise **w**_*k*_ and measurement noise **ν**_*k*_ are indeed Gaussian, the *a posteriori* estimate obtained in Eq (36) is optimal in the sense that it has minimum covariance **P**. In our case, the smartphone GPS derived distance and speed measurements are used to compare with and correct the *a priori* estimate of boat distance and speed.

The model prediction is performed at approximately 100 Hz in accordance with the sampling rate of the accelerometer, while the measurement update is carried out at the less frequent update rate of the GPS, about 0.3 Hz.

The performance of the Kalman filter relies heavily on the choice of values for the **Q** and **R** matrices. The optimal values for both are difficult, if not sometimes impossible, to know, and the noises are often not actually Gaussian. But in practice, the **Q** and **R** can often be tuned to create a good estimate. We are able to directly calculate the smartphone measurement variances by using the DGPS measurements as the true values (see Eqs (10) and (11)) and use them to populate the diagonals of **R**.

$$R=[\begin{array}{cc} 25.279 & 0\\ 0 & 100 \end{array}]$$ (40)

$$P_{0}=[\begin{array}{ccc} 1 & 0 & 0\\ 0 & 1 & 0\\ 0 & 0 & 0.51 \end{array}],\;x_{0}=[\begin{array}{c} d_{0}\\ v_{0}\\ 0.42 \end{array}]$$ (41)

Our process model is a simple and exact kinematic model so the only terms that may have appreciable process noise are the acceleration input and the bias. We assume that the process noise is negligible because of the quality of the acceleration measurement and the dominance of the bias term (over variance) in the development of error in the estimate. We thus, set **Q** = **0** to reflect this, and the filter trusts the model fully when no measurements are available, relying completely on the bias estimate to provide accurate estimates between measurement updates. The model is initialized with the first distance and speed measurement and an initial guess of the bias (see Eq 41 using the mean value raw acceleration, *α*_*y*,*k*_, for a single trial. It is noted that the initial state variance **P**_**0**_ are non-zero as we do not have confident knowledge about the initial values of states (including acceleration bias). Hence the measurements are used in the initial stage to adjust the state and bias estimation (even though **Q** = **0**), which is critical for achieving accurate estimation. We found the values for **Q** and **R** generally robust with respect to variations in rower and boat (see https://gitlab.com/mechmotum/row_filter for details). Note that we could obtain incremental gains in model accuracy if we further turned **Q** and **R** for individual rowers and boat configurations, however this choice would be inconsistent with our intent to build an easy-to-use, general purpose solution.

### A note on real-time algorithm implementation

We did not implement these filtering algorithms on an actual smartphone in real-time, but our algorithms, written in Python, can be directly translated to a smartphone’s associated programming language. The complementary filter, Kalman filter, and peak detection algorithms have 110, 440, and 160 floating point operations per time step, respectively. The maximum floating point operations per time step is then the sum of the Kalman filter and peak detection, 600. We desire real-time updates at 100 Hz so the total neccesary FLOPS is 60 thousand. Contemporary smartphones have FLOPS capabilities between 5 billion to 35 billion. Thus the real-time implementation of these algorithms is relatively trivial and has little consequence on overall computation time.

## Experimental methodology

**Fig 7:**
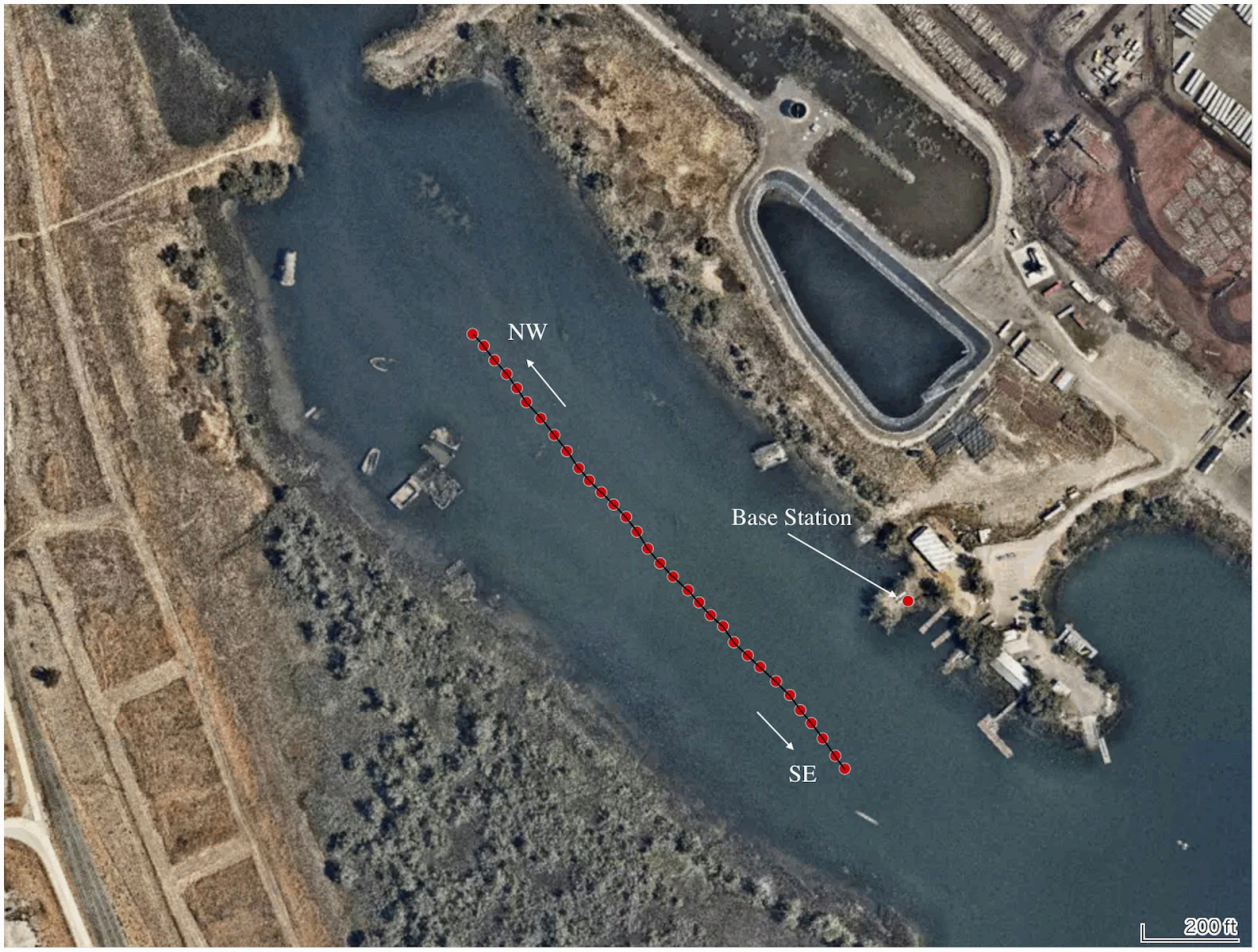
Satellite image showing a typical trial path. The single red dot on the shore is the location of the DGPS base station. The mean latitude and longitude are 38.566435°and -121.556365°, respectively.

Experiments were performed two days apart to validate the effectiveness of the proposed sensor fusion methods using a different rower-boat combination on each day: an experienced club-level (18 years rowing experience, age = 63, height = 1.68 m, weight = 70 kg) sculling a 2 person boat (2002 Hudson mid-weight, 2X) alone, and an elite rower (2016 Olympic participant, age = 31 height = 2.00 m, weight = 100 kg) sculling a single person boat (2004 Hudson heavy-weight, 1X). The 2X boat was used with a single rower to allow for ease of mounting of the measurement equipment to the empty bow seat before a mounting option for the single scull was developed. In each experiment, the rower performed a series of trials (each over a distance of approximately 300 m) in an inlet to a lake (Lake Washington, West Sacramento, CA, USA) that is part of deep water ship channel in both the northwest and southeast directions (Fig 7). A SpeedCoach GPS (Model 2, Nielson-Kellerman, Boothwyn, PA) was used onboard to display to the rower their current stroke rate. An example trial path is shown in Fig 7.

**Fig 8:**
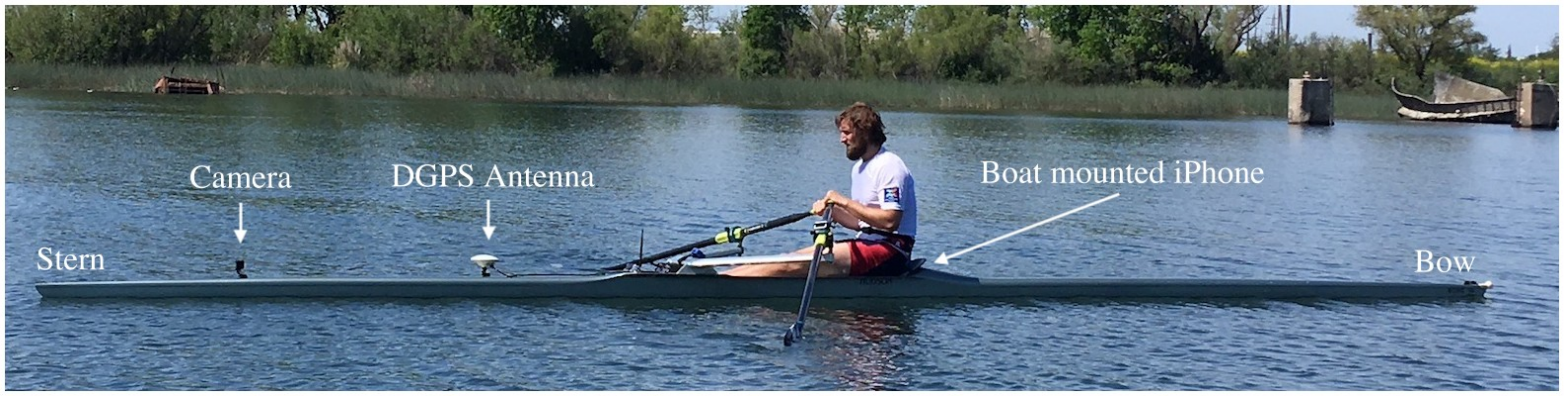
Sensor locations during elite trials. 2004 Hudson heavy-weight with the elite rower, annotated for experimental hardware. Note the DGPS antenna is clearly visible to the sky (above 15 degrees from horizontal in all directions it is with a clear view to the sky). The individual pictured has given written informed consent (as outlined in PLOS consent form) to publish these case details.

An iPhone 7 smartphone with iOS 11.3 (Apple, Cupertino, USA) running a custom data-logger app SwingRow 1.1 (Hegemony Technologies, Davis, CA) was rigidly attached to the deck of the 1X boat using positive-locking fasteners (Dual Lock, 3M, St. Paul, MN) at the position and orientation shown in Fig 8. A second smartphone running the same data-logger app was put into a “rowers wallet” (Hegemony Technologies, Davis, CA), positioned flat against the back of the rower at the top of the pelvis, and worn throughout the experiments. A ruggedized, waterproof camera (HERO4 Session, GoPro, San Mateo, USA) was mounted to the stern hull facing the rower to collect video. A differential GPS roving antenna (Piksi, Swiftnav, San Francisco, USA) was also attached to the hull as shown in Fig 8 (or in the spare seat in the case of the 2X boat).

Each rower performed a series of trials over a range of assigned stroke rates (target = 16, 20, 22, 24, 26, 28, max) in opposing directions (NW and SE) on the same 300 m course. The water current in the inlet was investigated and found to be negligible. The collected data is available in the supplementary materials.

The UC Davis IRB determined that this study is not research involving human subjects as defined by DHHS and thus IRB review was not required (IRB ID: 1430682-1). Informed consent was not formally obtained from the participants because it was not required under the IRB determination. The collected and shared data is anonymized and the portion of the data provided by Hegemony Technologies was anonymized before the authors’ analysis.

## Results

Both the smartphone and the DGPS provide time measurements originating from the same GPS satellites. We use these times to synchronize the measurements between devices and we calculate estimates of the three variables distance, speed, and distance per stroke at those times using the two aforementioned filters and directly from the smartphone position data. This section discusses the comparisons among these three estimates (smartphone: SP, complementary filter: CF, and Kalman filter: KF) of each of the variables. A description of the detailed analysis procedure can be found in the accompanying software (https://gitlab.com/mechmotum/row_filter). We present data summaries for each subject (rower-boat combination) in the following figures. We do so simply to show that the two filters are able to improve the metric estimates for subjects that have significant differences (mass, peformance, etc.) and purposely make no claims about filter performance between subjects due to having too few subjects.

### Filter convergence

**Fig 9:**
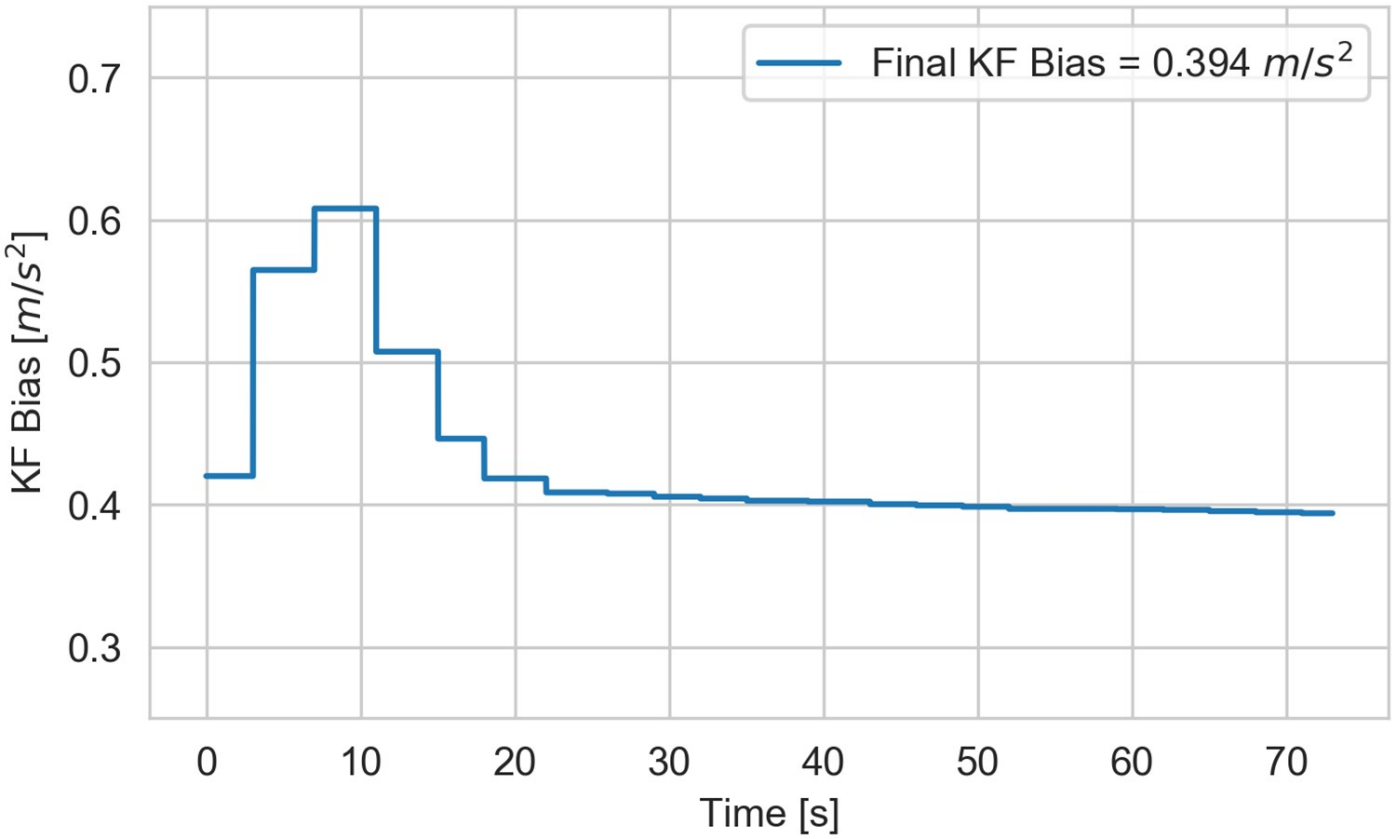
Convergence of the Kalman filter bias state over a single trial (elite 16NW). The filter bias state typically takes about 20 seconds to converge to a relatively constant value.

The Kalman filter’s performance relies on the effective bias *ϕ* converging to a constant value, because our model assumes the bias is constant. The state estimates will necessarily be erroneous if the bias is not constant in time. Fig 9 shows *ϕ* as a function of time for a single example trial. In this case it takes almost 20 seconds (or approximately 6 strokes) for convergence, which is about one fourth of the length of the trial. For this reason we limited the calculation of steady state performance data (RMSE) to the last ten strokes of each trial.

**Fig 10:**
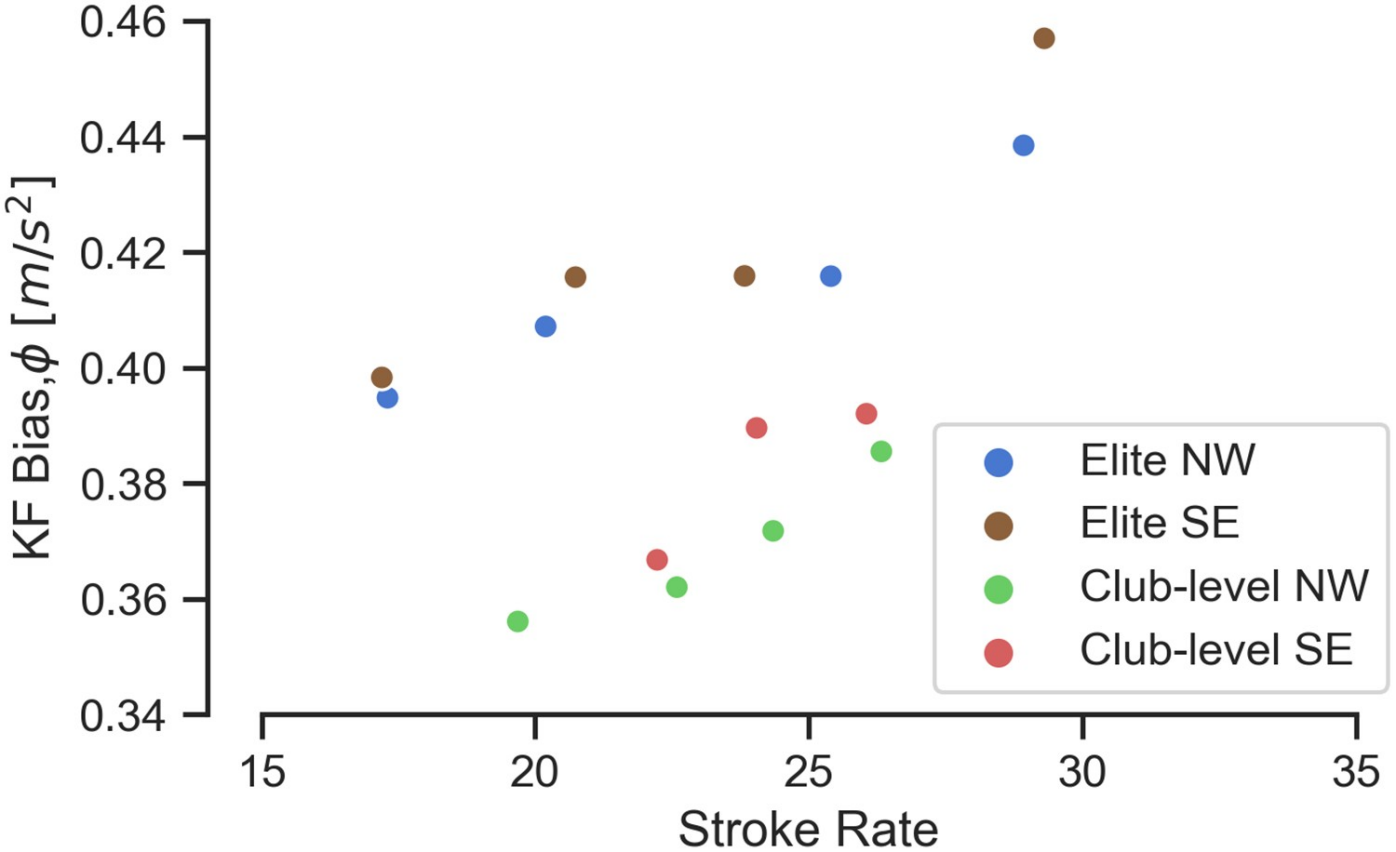
Terminal values of the Kalman filter bias state *ϕ*_*k*_ for each trial. Each dot represents a single trial at the mean stroke rate. The bias is dependent on the rower/boat combination and boat speed.

The filter converges to a different value of *ϕ* for each rower-boat combination and stroke rate. Fig 10 shows the steady state values of *ϕ* for every trial. The effective bias increases with stroke rate as does the average boat pitch angle.

Rowing races at amateur and professional levels typically range from 1000-5000 meters in length and are completed in timeframes that range from 3 minutes to 20+ minutes. Every boat before a race will execute an extensive warmup involving many hundreds and probably thousands of strokes over 30+ minutes. This warmup period provides ample opportunity to complete all of the filter convergence for this implementation so that it will be optimally tuned and operational for the totality of a race.

### Distance estimates

**Fig 11:**
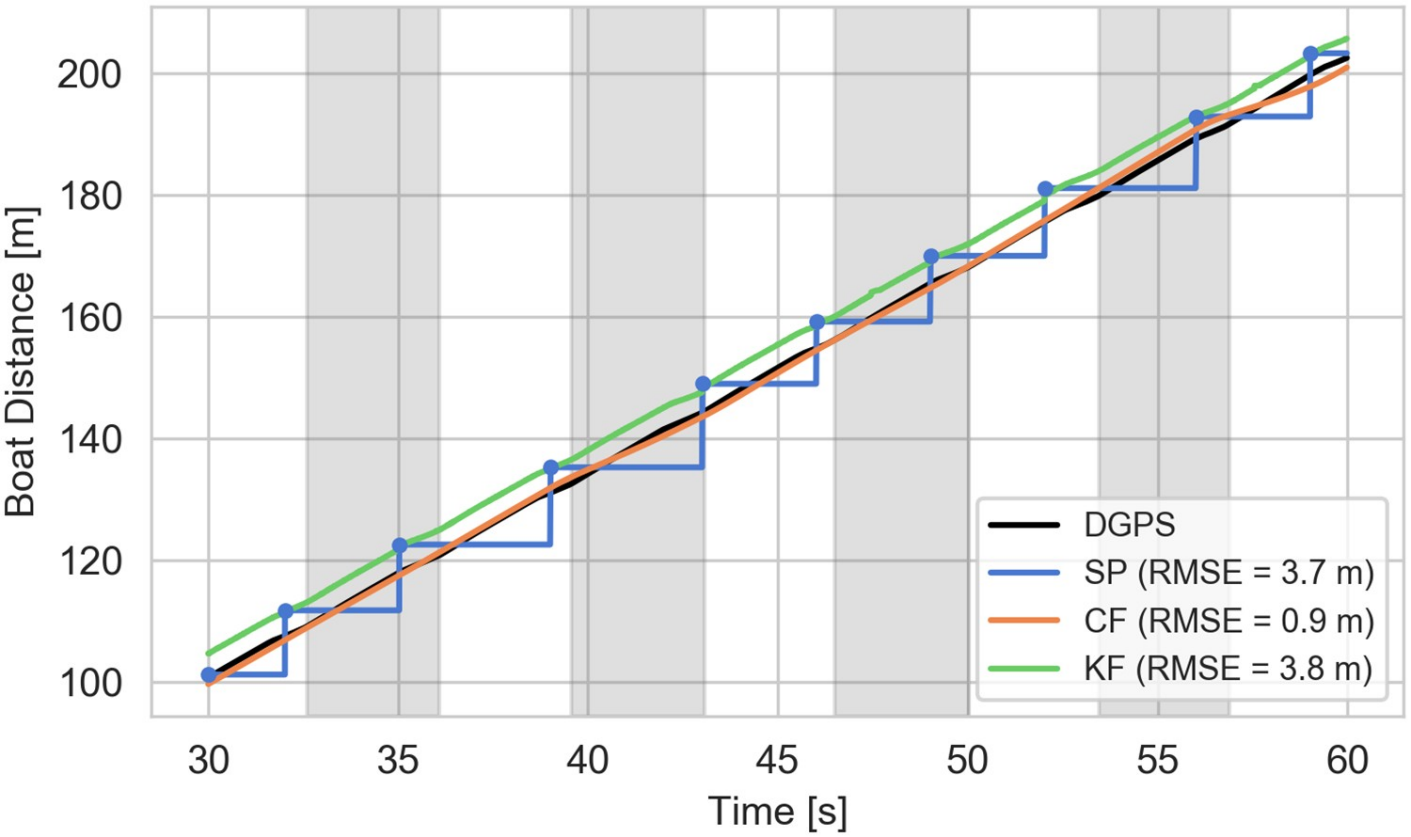
Example total boat distance traveled estimates. The figure indicates distance traveled as a function of time for the last 30 s of the elite 16NW trial as estimated by the smartphone, complementary filter, and Kalman filter. The blue dots indicate the actual smartphone measurement update and the blue line is the piecewise constant interpolation in between updates. The alternating gray and white sections indicate each stroke. The reported RMSE values are relative to the DGPS distance shown in black. Each RMSE is calculated at the accelerometer sampling rate, i.e. 100 Hz.

**Fig 12:**
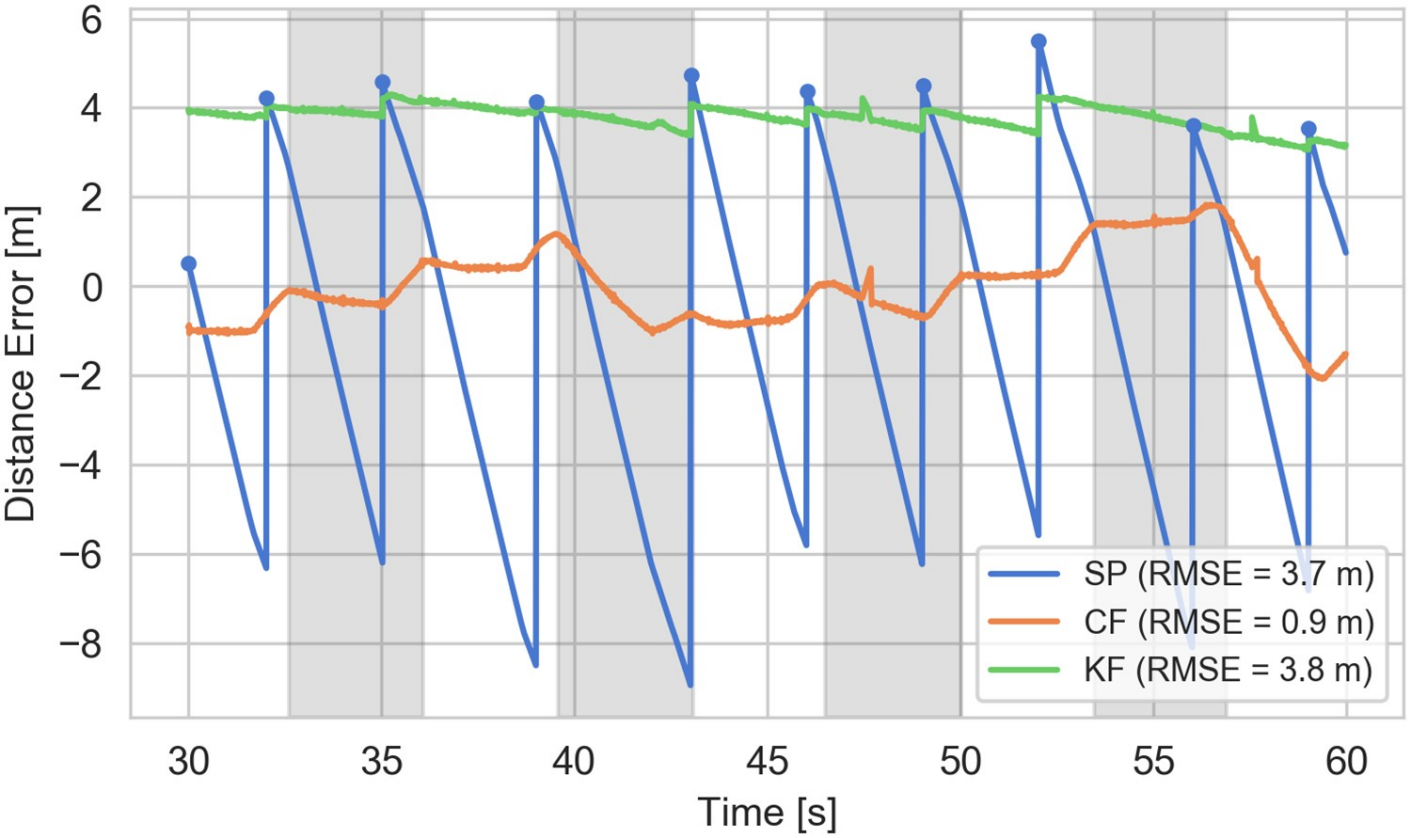
Example boat distance estimate errors. Error in the smartphone, complementary filter, and Kalman filter distance estimates relative to the DGPS for the last 30 s of the elite 16NW trial. The “saw-tooth” smartphone error curve is derived from the piece-wise constant curve shown in Fig 11 to highlight the issue it poses when one desires to calculate distance per stroke. The alternating gray and white sections indicate each stroke. The RMSE values are the same as in Fig 11.

Fig 11 shows, using an example from a single elite trial (16NW) after filter convergence, all estimates of total distance travelled. Fig 12 shows the errors of these estimates relative to the DGPS-derived distance. The Kalman filter estimate is similar to the smartphone at the GPS updates and provides a reasonably drift free estimate between adjacent smartphone updates; that is, it is similar in accuracy to the smartphone but much more precise. The complementary filter is less influenced by the smartphone distance measurements and provides a better estimate of the true distance traveled, both with respect to accuracy and precision. This is because the complementary filter corrects the integration bias solely from the speed measurement and mostly ignores the position measurement.

**Fig 13:**
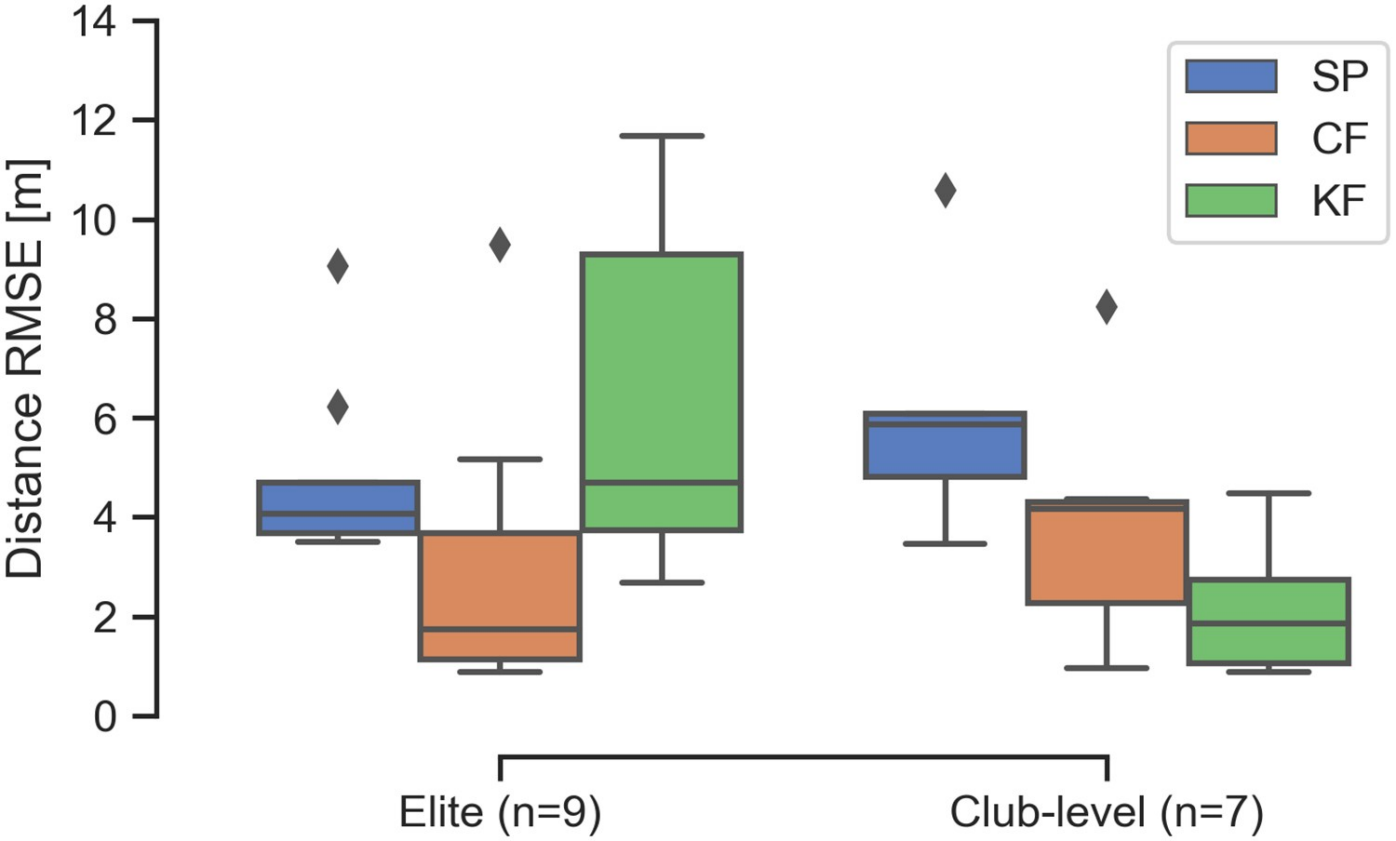
Summary of the distance estimate errors for all trials. Comparisons of the distributions of RMSE_*d*_ of the distance estimates from the three methods for the last 10 strokes of each trial.

Fig 13 portrays the distribution of RMSE for the distance estimates relative to the DGPS for all trials for the elite and club-level rowers. The complementary filter shows improvement for both rowers and the Kalman filter shows improvement for the club-level rower. The Kalman filter actually is more than a meter worse for the elite rower when comparing the medians. The large distance RMSE for the filters is attributable to the relatively poor accuracy in the GPS measurement. In contrast, the errors in distance per stroke estimates are primarily influenced by measurement precision, which are improved by the filters relative to the smartphone. Nevertheless, the complementary and Kalman filters improve the median estimate by 42% and 22% when all trials are considered.

### Boat speed estimates

**Fig 14:**
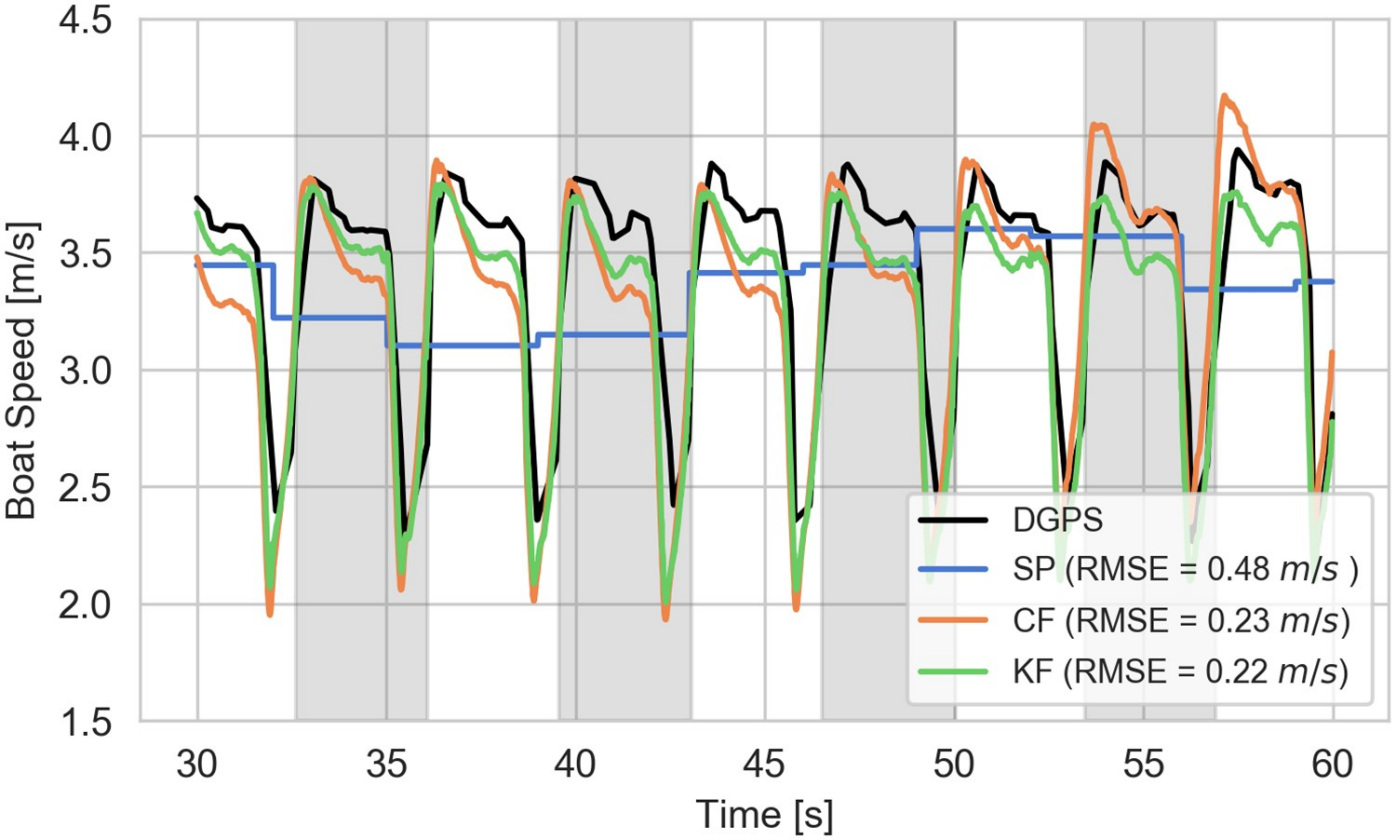
Example boat speed estimates. The figure indicates the speed as a function of time for the last 30 s of the elite 16NW trial as estimated by the smartphone, complementary filter, and Kalman filter. The alternating gray and white sections indicate each stroke. The reported RMSE values are relative to the DGPS computed speed shown in black. Each RMSE_*v*_ is calculated at the 100 Hz accelerometer sampling rate.

Fig 14 shows example speed estimates for a typical trial after convergence from both the complementary and Kalman filters compared to those derived from the raw smartphone GPS and the differential GPS measurements. The RMSE of the estimates relative to differential GPS are tabulated for the post-convergence portion and shown on the graph for that trial. Both filters track the differential GPS derived speed throughout the stroke much more closely than the smartphone GPS derived speed, which is more like an average speed. Both of the filters improve the estimate by over a factor of 2 in this trial.

**Fig 15:**
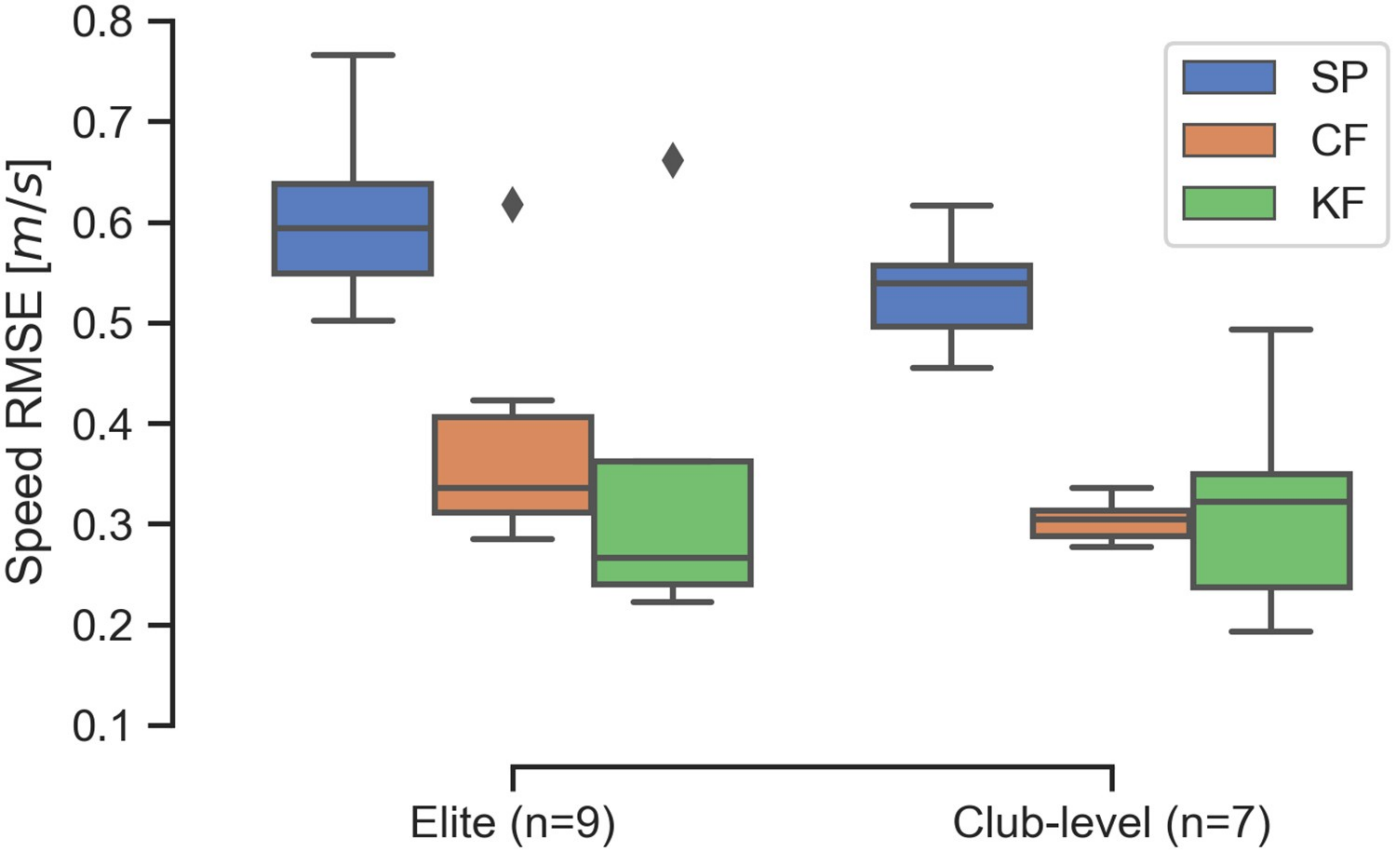
Summary of the speed estimate error for all trials. Comparisons of the distributions of RMSE_*v*_ of the speed estimates from the three methods for the last 10 strokes of each trial.

Fig 15 shows the summary of the calculated speed RMSEs for each rower. It is clear that both of the filters improve the speed estimates for all trials, also by about a factor 2 or more when comparing the medians. Overall, the complementary and Kalman filters improve the median estimate by 44% and 48% when all trials are considered.

### Distance per stroke estimates

**Fig 16:**
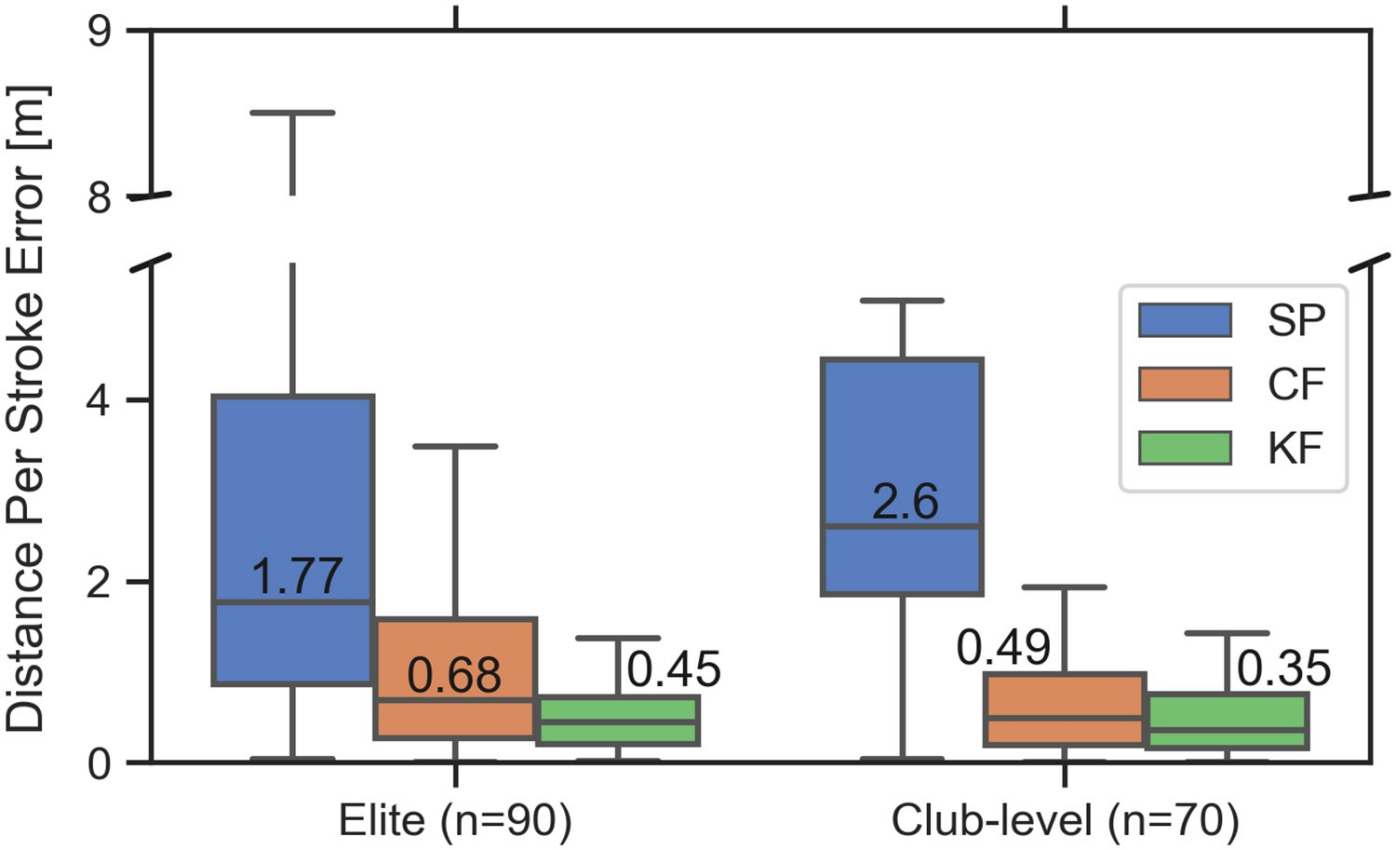
Summary of the distance per stroke error for the last 10 strokes of each trial. Comparison of the distance per stroke error relative to the DGPS-derived value for all trials for the elite and club-level rowers, respectively. The numbers indicate the median values.

Fig 16 compares the distance per stroke estimates computed from the smartphone, complementary filter, and Kalman filter with respect to the differential GPS derived estimates. The percentage improvement for the complementary filter is 62% and 81% for the elite and club-level rowers whereas for the Kalman filter it is 75% and 87%, respectively. The average of the error median values of the filters is 49 cm, which is still an order of magnitude larger than the goal of less than 5 cm. It is important to note that distance per stroke estimates are not affected by any constant bias present in the distance error (Fig 12). As long as the distance estimate has good precision and equivalent slope to the actual distance traveled across each stroke, the distance per stroke errors can be low.

## Discussion

Rowing research, training, and racing methodologies are necessarily linked to the accuracy and precision of the available measurement systems. The emergence of location technologies like GPS make it possible to derive and report speed and related metrics in the stroke domain, e.g., stroke rate and distance per stroke. However, efforts to monitor and effect meaningful elite-level race result at an individual stroke level were shown in the introduction to require location accuracy and precision better than 5 cm, which systems like GPS cannot deliver. Thus, the aforementioned stroke level metrics derived from available systems should be recognized as approximations. These instrumentation limitations prevent direct and quantitative investigations of the complex causal relationships between rower-oar-boat system mechanics and boat performance at and within the level of an individual stroke. For these purposes, this study has explored methods for achieving more accurate and precise measures of boat movement. We created a system using consumer electronics and services and focused on designing a general purpose and easy-to-use solution that could be broadly deployed in the rowing community.

We have presented two alternative estimation methods for boat distance traveled, boat speed, and distance per stroke. Both methods similarly perform better (are more accurate) in most cases than direct output from the smartphone, but neither reach the desired sub 5 cm distance per stroke accuracy. The complementary filter has the disadvantage that the filter cutoff frequencies were not updated to optimal values in real-time, and the optimal offline values we found do not robustly handle all stroke rates for the two rowers and boats investigated. This makes the Kalman filter method more attractive because the bias term is adaptively updated for every rower and boat; i.e. the filter tunes itself. Nevertheless the complementary filter performs as well as or better than the Kalman filter for our set of trials. Both filters take time to converge to a steady error from a zero speed start, so the first few strokes in a race will produce less accurate results. A future study could consider minimizing the startup time by tuning the filters further, but there is likely a tradeoff in accuracy and precision of the estimates.

Both of our presented methods provide better estimates of boat speed and distance per stroke over any prior work that uses a single low-cost commercially available GPS system. The closest prior work on rowing is the thesis from Hermsen [21]. Hermsen’s concept was similar but did not offer the online adaptation that our Kalman filter design provides and there were no reported improvements in any metric but predicted time. Our methods do not provide estimates as accurate as the measurements available using differential GPS systems, but considering the cost and convenience of use our methods are more attractive for general consumer use cases.

We have sought to develop a general purpose boat motion model that is independent of stroke rate, and the models presented in this paper were constructed from experiments involving single rowers with rowing rates that ranged from 16 to 34 strokes per minute. The markedly inferior performance of distance per stroke measures in the smartphone (SP) estimate relative to the CF and KF methods is largely attributed to the smartphone’s limitation of having only a relatively low position sampling frequency. In the case of high stroke rate rowing where stroke frequency (at the high end faster than 0.5 Hz) exceeds the smartphone location sampling frequency (0.3–0.5 Hz), there are numerous instances between location samples where a stroke ends, a second stroke is completed, and a third stroke begins. In these cases, the distance per stroke error of the second stroke is the entire distance traveled. Accordingly, the CF and KF models stand to add the most value for high rate rowing, e.g., racing rates.

It may be possible to further reduce estimation errors by implementing changes to the model that reflect unique or special aspects of rowing. For example, rowing necessarily occurs on a level plane (water) and boat movement dominantly occurs along the longitudinal axis of the boat. Both of these conditions imply kinematic constraints that were not completely modeled in this study. If the choice to build a general-purpose model was relaxed and special purpose models were developed that were tuned to specified ranges of rowing rates, rowing ability, and boat class, then special purpose model accuracies would definitely improve. Additionally, relatively expensive commercial sports position and speed sensors can sample position at higher rates than a smartphone and thus can be useful to address the significant errors due to the low frequency sampling of the smartphone. However, these high frequency sampling solutions do not eliminate the inaccuracies and imprecision of the position measurements and thus at present do not represent a viable method for realizing the distance per stroke estimation accuracy of 5 cm or less. Rowers and teams could also invest in a DGPS and immediately gain the necessary accuracy and precision, but the costs are higher and the hardware is more cumbersome. Once we accomplish improvements that can achieve this level of accuracy and thus can enable more microscopic analyses of rowing mechanics, we anticipate the emergence of a new generation of tools for testing and coaching the boat-racing performance.

## Conclusion

We have presented two methods to estimate the distance, speed, and distance per stroke along a rowing boat’s path in real time that provide improved accuracy and precision results from the relatively low accuracy sensors in a single smartphone attached to the boat. These improved estimates can be used to create a more detailed analysis of the rower’s performance. Specifically, we show that the distance per stroke can be estimated to an accuracy and precision of about 50 cm, which is an improvement over smartphone estimates but still insufficient for detailed stroke-by-stroke level differentiation of boats in a racing event with relatively close elapsed times. The more continuous data on boat speed that our methods create open up opportunities to analyze rowing mechanics and performance *within* a stroke. Overall, this paper demonstrates the capability that carefully crafted, activity-specific sensor fusion algorithms can have with low accuracy sensors. Accessible inertial measurement units, like those in smartphones, are continually decreasing in cost and size and stand to play a larger role in collecting field data in sports. The utility of these systems will depend on the development and improvement of application-specific sensor fusion algorithms.

## Nomenclature

### Complementary filter

**Table:** 

*ω*	frequency
*ω*_*c*_	cutoff frequency
*A*	smartphone accelerometer input signal
*a*_0_	accelerometer bias
*D*	smartphone GPS input signal
*H*	transfer function
*j*	unit imaginary number
*k*	index for prior GPS update
*s*	denotes the frequency domain (*s* = *jw*)
*V*	smartphone speed input signal
*X*	input signal
*Y*	output signal

### Error calculations

**Table:** 

${\bar{d}}_{e},{\bar{v}}_{e}$	mean errors of distance and speed estimates
$\bar{x},\bar{y}$	mean of Cartesian coordinates
$\sigma_{d_{e},v_{e}}$	standard deviation of distance and speed estimate errors
*σ*_*x*,*y*_	standard deviation of the Cartesian coordinates
${\text{CE}}_{d_{e},v_{e}}$	central errors of the distance and speed estimates
CE_*xy*_	central errors of the Cartesian location
${\text{RMSE}}_{d,v,d_{s}}$	root mean square errors of distance, speed, and distance per stroke
RMSE_*xy*_	root mean square errors of the Cartesian location
*d*	distance traveled along the boat’s path
*d*_*e*_, *v*_*e*_	errors in distance and speed estimates, respectively, relative to differential GPS measurements
*d*_*s*_	distance per stroke
*i*	sample index
*m*	Number of strokes
*n*	number of time samples
*t*	time
*v*	magnitude of the velocity along the boat’s path
*x*	east-west position on the local EPSG 3310 WGS84 plane
*x*_*s*_, *y*_*s*_	true Cartesian coordinates
*y*	north-south position on the local EPSG 3310 WDGS84 plane

### Kalman filter

**Table:** 

*α*_*yk*_	input: smartphone body-fixed longitudinal acceleration component
***ν***_*k*_	measurement noise vector
Δ*t*	time differential
**A**	state transition matrix
**B**	input matrix
**C**	output matrix
**D**	feed-through matrix
**L**_*k*_	gain matrix
**P**_0_	initial estimate covariance
**P**_*k*_	estimate covariance
**Q**	process noise covariance matrix
**R**	measurement noise covariance matrix
**u**_*k*_	input vector
**w**_*k*_	process noise vector
**x**_0_	initial state vector
**x**_*k*_	state vector
**y**_*k*_	output vector
*ϕ*_*k*_	state: accelerometer bias
*a*_*k*_	input: acceleration
*d*_*k*_	state: distance
*v*_*k*_	state: speed

### Other symbols

**Table:** 

*α*_*x*,*y*,*z*_	smartphone body-fixed acceleration components
***α***	boat mounted body-fixed smartphone acceleration vector
${\hat{x}}^{\prime },{\hat{y}}^{\prime },{\hat{z}}^{\prime }$	smartphone path-fixed unit vectors
$\hat{x},\hat{y},\hat{z}$	smartphone body-fixed unit vectors
**a**	path-fixed acceleration vector
*θ*	boat pitch angle
*a*	magnitude of the acceleration along the boat’s path
*a*_*x*,*y*,*z*_	smartphone path-fixed acceleration components
*g*	acceleration due to gravity
CF	abbreviation for complementary filter
KF	abbreviation for Kalman filter
SP	abbreviation for smartphone
